# PI(4)P Promotes Phosphorylation and Conformational Change of Smoothened through Interaction with Its C-terminal Tail

**DOI:** 10.1371/journal.pbio.1002375

**Published:** 2016-02-10

**Authors:** Kai Jiang, Yajuan Liu, Junkai Fan, Jie Zhang, Xiang-An Li, B. Mark Evers, Haining Zhu, Jianhang Jia

**Affiliations:** 1 Markey Cancer Center, University of Kentucky College of Medicine, Lexington, Kentucky, United States of America; 2 Department of Pediatrics, University of Kentucky College of Medicine, Lexington, Kentucky, United States of America; 3 Department of Surgery, University of Kentucky College of Medicine, Lexington, Kentucky, United States of America; 4 Department of Molecular and Cellular Biochemistry, University of Kentucky College of Medicine, Lexington, Kentucky, United States of America; Stanford University School of Medicine, UNITED STATES

## Abstract

In Hedgehog (Hh) signaling, binding of Hh to the Patched-Interference Hh (Ptc-Ihog) receptor complex relieves Ptc inhibition on Smoothened (Smo). A longstanding question is how Ptc inhibits Smo and how such inhibition is relieved by Hh stimulation. In this study, we found that Hh elevates production of phosphatidylinositol 4-phosphate (PI(4)P). Increased levels of PI(4)P promote, whereas decreased levels of PI(4)P inhibit, Hh signaling activity. We further found that PI(4)P directly binds Smo through an arginine motif, which then triggers Smo phosphorylation and activation. Moreover, we identified the pleckstrin homology (PH) domain of G protein-coupled receptor kinase 2 (Gprk2) as an essential component for enriching PI(4)P and facilitating Smo activation. PI(4)P also binds mouse Smo (mSmo) and promotes its phosphorylation and ciliary accumulation. Finally, Hh treatment increases the interaction between Smo and PI(4)P but decreases the interaction between Ptc and PI(4)P, indicating that, in addition to promoting PI(4)P production, Hh regulates the pool of PI(4)P associated with Ptc and Smo.

## Abstract 2

The signaling molecule Hedgehog promotes the production and release of the specific phospholipid PI(4)P, which directly interacts with Smoothened, activating it via phosphorylation and accumulation in the cilia.

## Author Summary

The Hedgehog (Hh) signaling pathway plays important roles in both embryonic development and adult tissue homeostasis. A critical step in Hh signal transduction is the inhibition of Smoothened (Smo), an atypical G protein-coupled receptor (GPCR), by the Hh receptor Patched (Ptc). It is a longstanding question how Ptc inhibits Smo and how Hh promotes Smo phosphorylation and activation. It is unlikely that Ptc inhibits Smo by direct interaction. Here, we uncover that phosphatidylinositol 4-phosphate (PI(4)P), a specific phospholipid, directly interacts with Smo through an arginine motif in the Smo C-terminal tail and promotes Smo phosphorylation, activation, and ciliary localization. Ptc also interacts with PI(4)P, which is inhibited by Hh stimulation, indicating that Hh triggers the release of PI(4)P from Ptc. We further uncover that Hh stimulation induces the overall production of PI(4)P, likely by regulating PI(4)P kinase and phosphatase. Finally, in addition to the direct role in regulating Smo phosphorylation, G protein-coupled receptor kinase 2 (Gprk2) facilitates PI(4)P interaction with Smo. This study suggest that PI(4)P acts as a special small molecule shuttling between Ptc and Smo to modulate Hh responses.

## Introduction

The Hedgehog (Hh) signaling pathway plays important roles in both embryonic development and adult tissue homeostasis [1–3]. In *Drosophila*, the Hh signal is transduced through a receptor system at the plasma membrane, which includes the receptor complex Patched-Interference Hh (Ptc-Ihog) and the signal transducer Smo [4–6]. Binding of Hh to Ptc-Ihog relieves the Ptc-mediated inhibition of Smo, which allows Smo to activate the cubitus interruptus (Ci)/Gli family of zinc finger transcription factors and thereby induce the expression of Hh target genes such as *decapentaplegic* (*dpp*), *ptc*, and *engrailed* (*en*) [7,8]. Over the last 30 years, many Hh pathway components have been identified, including those that control transmission, propagation, receipt, and transduction of the Hh signal. However, it is still unclear how Ptc inhibits Smo to block the activation of the Hh pathway and how Ptc inhibition of Smo is relieved by Hh stimulation. It is unlikely that Ptc inhibits Smo by direct association [9,10], as the inhibition occurs even when Smo is present in 50-fold molar excess of Ptc, and substochiometric levels of Ptc can repress Smo activation [10,11]. These findings suggest that the inhibition process is catalytic [10]. The involvement of small molecules, rather than a protein ligand, has been proposed: Ptc may inhibit the production of positive regulators or promote the synthesis of inhibitory molecules [10].

Smo, an atypical G protein-coupled receptor (GPCR), is essential in both insects and mammals for transduction of the Hh signal [8,12,13]. The activation of Smo appears to be one of the most important events in Hh signaling. Hh induces cell surface accumulation and phosphorylation of Smo [9] by multiple kinases, including protein kinase A (PKA), casein kinase 1 (CK1) [14–16], casein kinase 2 (CK2) [17], G protein-coupled receptor kinase 2 (Gprk2) [18], and atypical PKC (aPKC) [19]. These phosphorylation events activate Smo by inducing a conformational change [20] to promote Smo interaction with the Costal2-Fused (Cos2-Fu) protein complex [21–23]. It is believed that Hh-induced phosphorylation counteracts the autoinhibition imposed by arginine clusters in the Smo C-terminal tail (C-tail), which induces an open conformation that promotes the dimerization of Smo proteins [1,20]. Similar to other GPCRs, Smo cell surface accumulation is controlled by endocytic trafficking mediated by ubiquitination [24,25]. In mammals, Hh signal transduction depends on the primary cilium, and Smo accumulation in the cilium is required for Smo activation [26–28]. Therefore, the cilium represents a signaling center for the Hh pathway in mammals [29]. Phosphorylation by multiple kinases promotes the ciliary localization of mammalian Smo [30], but it remains unclear how Smo cell surface or ciliary accumulation and intracellular trafficking are controlled. A previous study has shown that mutation in INPP5E, a lipid 5-phosphotase, results in signaling defects in primary cilium [31], indicating a role for phospholipids to regulate the function of cilium. In the preparation of this manuscript, two studies found that phospholipids regulate ciliary protein trafficking [32,33]; however, it is unknown whether and how phospholipids directly regulate Smo.

A very well characterized system for studying Hh signaling is the *Drosophila* wing disc. Hh proteins expressed and secreted from the posterior (P) compartment cells act on neighboring anterior (A) compartment cells located adjacent to the A/P boundary to induce the expression of Dpp [34,35]. As a morphogen, Dpp diffuses bidirectionally into both the A and P compartments to control the growth and patterning of cells in the entire wing [36–38]. Other genes, including *en* and *ptc*, are also induced by Hh to specify cell patterning at the A/P boundary [39,40]. Expression of *dpp* monitors the low levels of Hh activity, and *ptc* expression indicates higher levels of Hh activity, whereas *en* induction appears to be an indicator of the highest doses of Hh signaling activity [39]. The transcription factor Ci is only expressed in A compartment cells that receive the Hh signal.

In this study, we found that Hh stimulation increases the levels of phosphatidylinositol 4-phosphate (PI(4)P) in both wing discs and cultured cells. We further found that PI(4)P activates Smo by promoting Smo phosphorylation. Mechanistically, we identified an arginine motif in the Smo C-tail that is responsible for the interaction of Smo with PI(4)P. Arginine to alanine mutation abolishes, whereas arginine to glutamic acid mutation elevates, Smo activity. We also found that, in addition to the kinase activity of Gprk2, its pleckstrin homology (PH) domain increases PI(4)P in wing discs and is required for Gprk2 to fully function in Hh signaling. The findings that Ptc interacts with PI(4)P and that Ptc inactivation increases the levels of PI(4)P indicate that PI(4)P acts downstream of Ptc to activate Smo in the Hh signaling cascade. Finally, we show that PI(4)P promotes phosphorylation and ciliary accumulation of mouse Smo (mSmo) in mammalian cells, and that PI(4)P prevents the ciliary accumulation of mouse Ptc1 and Ptc2. Taken together, our findings suggest that PI(4)P acts as a special small molecule shuttling between Ptc and Smo to modulate Hh responses.

## Results

### Hh Promotes the Production of PI(4)P

In an effort to identify novel regulators in Hh signaling, we collected RNAi lines from the Vienna *Drosophila* Resource Center (VDRC) when the library was available and screened for kinases, phosphatases, and E3 ubiquitin-protein ligases using the wing-specific *MS1096*-Gal4. We tested selected RNAi lines for the ability to modify the phenotype of Smo^PKA12^, a weak dominant negative form of Smo that results in a reproducible wing phenotype with partial fusion between Vein 3 and Vein 4 when combined with the *C765*-Gal4 (S1D Fig), which represents a very sensitive genetic background for screening Smo regulators [19,25,41]. One of the “hits” was Stt4 kinase, the yeast homolog of PI4KIIIalpha required for the generation of PI(4)P. We found that, although knockdown of Stt4 alone did not produce any change in the wild-type wing (S1B Fig), Stt4 RNAi combined with Smo^PKA12^ expression enhanced the fusion of Vein 3 and Vein 4 (S1E Fig). In contrast, inactivation of Sac1 phosphatase, which dephosphorylates PI(4)P to phosphatidylinositol (PI), attenuated the fusion phenotype (S1F Fig). These results suggest that Stt4 and Sac1 regulate the activity of Smo in the wing. Consistently, Sac1 RNAi partially rescued the abdominal cuticle loss caused by Hh RNAi, although Sac1 RNAi alone did not show any cuticle phenotype (S1G–S1J Fig). A recent study established a genetic link between Smo and Stt4-Sac1 [42]; however, the molecular mechanisms are unclear.

**Fig 1:**
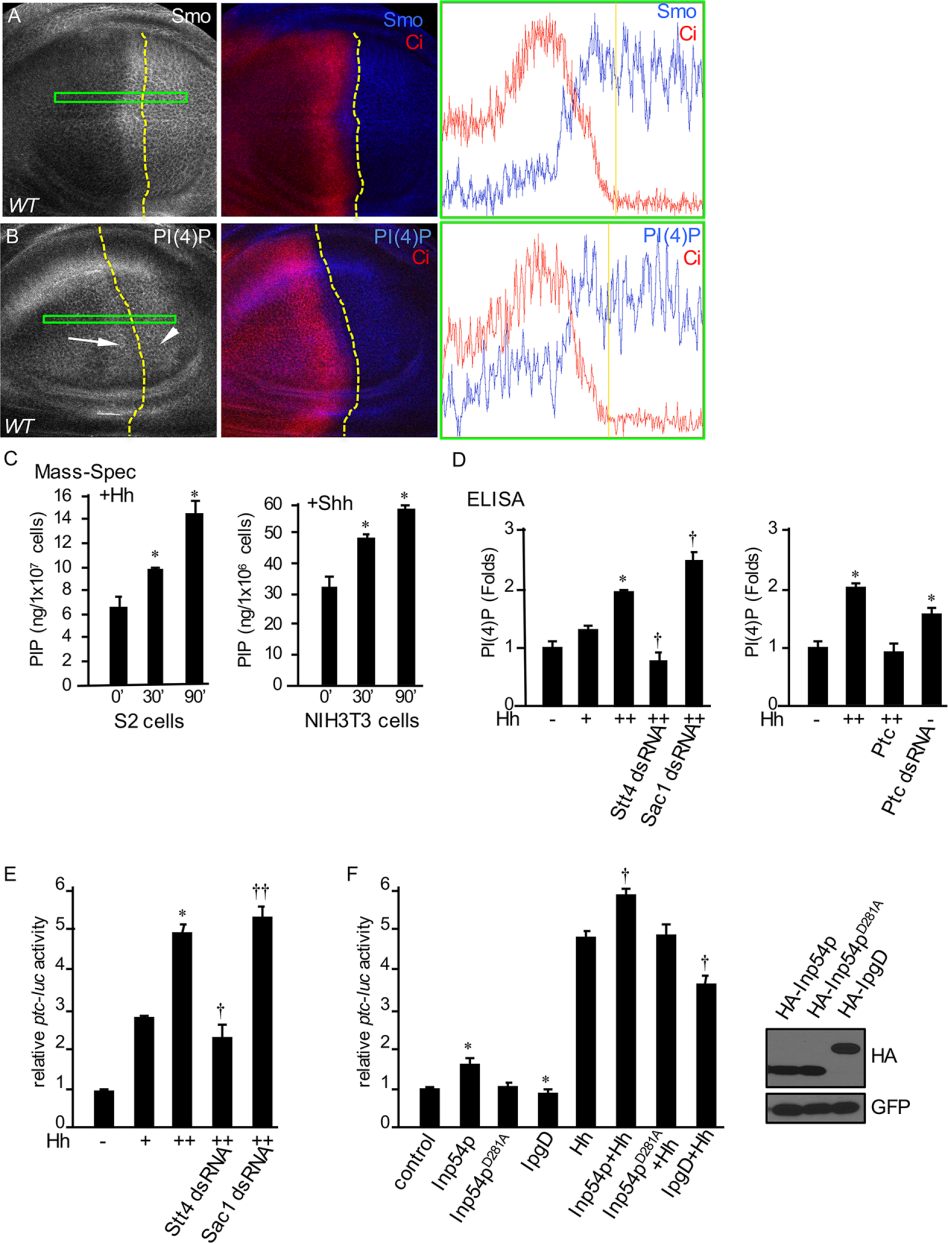
Hh promotes the production of PI(4)P. (A) A WT disc was stained for Smo. Dashed yellow line indicates the A/P boundary defined by Ci staining. Green box indicates the region for density analysis that is shown on the right, with a yellow line indicating the A/P boundary. (B) A WT disc was stained for Ci and PI(4)P. Arrow indicates PI(4)P accumulation in A compartment cells near the A/P boundary, and arrowhead indicates PI(4)P accumulation in P compartment cells. Dashed yellow lines indicate the A/P boundary defined by Ci staining. Green box indicates the region for density analysis using ImageJ software (NIH, version 1.48v) that is shown on the right with a yellow line indicating the A/P boundary. All wing imaginal discs shown in this study were dissected from third instar larvae with different genotypes and are shown with anterior on the left and ventral on the top. (C) Effect of Hh treatment on PIP content. S2 or NIH3T3 cells were stimulated with either 60% Hh-conditioned medium or Shh protein (5nM) before lipid extraction. A Thermo TSQ Vantage triple-quad mass spectrometer coupled with a Shimadzu HPLC as the front-end separation was used for the MRM measurement. * *p* < 0.01 versus time 0 (Student’s *t* test). (D) Left panel: S2 cells were treated with Stt4 dsRNA or Sac1 dsRNA, followed by treatment with control medium, 30% Hh-conditioned medium (Hh^+^), or 60% Hh-conditioned medium (Hh^++^). PI(4)P was detected by ELISA. * *p* < 0.01 versus control (first column); † *p* < 0.01 versus high level of Hh treatment (third column). Right panel: S2 cells were transfected with UAST-Ptc construct or treated with Ptc dsRNA and PI(4)P detected by ELISA. *p* < 0.01 versus control (first column). (E) S2 cells were treated with the indicated dsRNA followed by treatment with different amounts of Hh-conditioned medium and assayed for *ptc*-luc reporter activity. * *p* < 0.01 versus control (first column). † *p* < 0.01 versus high level of Hh (third column). †† *p* < 0.05 versus high level of Hh (third column). The efficiency of RNAi is shown in S3C and S3D Fig. (F) S2 cells were transfected with indicated constructs, treated with 60% Hh-conditioned medium, and assayed for *ptc*-luc activity. * p < 0.05 versus control. † *p* < 0.001 versus Hh alone. In these experiments, S2 cells stably expressing *tub-*Ci were used as they have full responsiveness to Hh stimulation. The expression of HA-Inp54p, Inp54p^D281A^, and IpgD are shown in the right panel by direct western blot with the anti-HA antibody, using lysates from cells expressing the indicated HA-tagged constructs. GFP was used as a transfection and loading control. The underlying data of panels C–F can be found in S1 Data.

Smo accumulates in P compartment cells as well as A compartment cells near the A/P boundary (Fig 1A) [9,15]. We found that the level of PI(4)P is elevated in the A compartment cells that abut the A/P border (Fig 1B), suggesting that Hh induces the accumulation of both Smo and PI(4)P in these cells. Consistently, the accumulation of Smo in *Drosophila* embryo correlated with the accumulation of PI(4)P (S2A–S2C Fig). We further found that PI(4)P levels were increased by the expression of Ci^-3P^ and Smo^SD123^, the constitutively active forms of Ci and Smo, respectively (S2D and S2E Fig). To accurately measure and quantify the absolute concentration of PIP in cells, we established a mass spectrometry-based multiple reaction monitor (MRM) assay and examined whether Hh indeed induces the production of PIP. Based on a detailed method published recently for quantifying PIP_2_ (PI(3,4)P_2_, PI(3,5)P_2_, PI(4,5)P_2_), and PIP_3_ (PI(3,4,5)P_3_) [43], we optimized the conditions to examine PIP lipids. Trimethylsilyl diazomethane was used to protect the phosphate groups, which allowed for more efficient ionization of the methylated PIP species and a marked improvement in the sensitivity of the assay. We found that treatment of S2 cells with 60% Hh-conditioned medium [44] induced the formation of PIP in a timely manner (Fig 1C, left panel). Consistent with this, treatment of NIH3T3 mouse fibroblasts with mouse sonic Hh N-terminus (ShhNp) [30] stimulated the production of PIP (Fig 1C, right panel). Total PIP was quantified, since this assay was unable to distinguish PI(4)P from PI(3)P and PI(5)P.

To further characterize the regulation of PI(4)P by Hh, we used an enzyme-linked immunosorbent assay (ELISA) and found that Hh stimulated the production of PI(4)P in S2 cells in a concentration-dependent manner (Fig 1D, left panel). In addition, knockdown of Stt4 downregulated, whereas knockdown of Sac1 upregulated, the production of PI(4)P (Fig 1D, left panel), suggesting that the Hh-regulated formation of PI(4)P was mediated by Stt4 and Sac1. We further found that overexpression of Ptc prevented the production of PI(4)P, whereas RNAi-mediated knockdown of Ptc elevated production (Fig 1D, right panel), suggesting that Ptc regulates the levels of PI(4)P. To delineate the involvement of PI(4)P in Hh signaling, we used a *ptc-*luciferase (*ptc-*luc) reporter assay to monitor the activity of Hh signaling [44] and found that Hh-induced *ptc*-luc activity was suppressed by RNAi of Stt4 but elevated by RNAi of Sac1 (Fig 1E). Furthermore, treatment with PI(4)P and the expression of Inp54p (a PI(4,5)P_2_-specific phosphatase to produce PI(4)P) enhanced, whereas IpgD (converts PI(4,5)P_2_ into PI(5)P) suppressed, the basal and Hh-induced *ptc*-luc activity (Figs 1F and S3A). As a control, Inp54p^D281A^ phosphatase-dead mutant had no effect on *ptc*-luc activity (Fig 1F). These data suggest that PI(4)P is a specific phospholipid that regulates Hh signaling in cultured S2 cells.

### PI(4)P Stimulates the Phosphorylation and Accumulation of Smo

**Fig 2:**
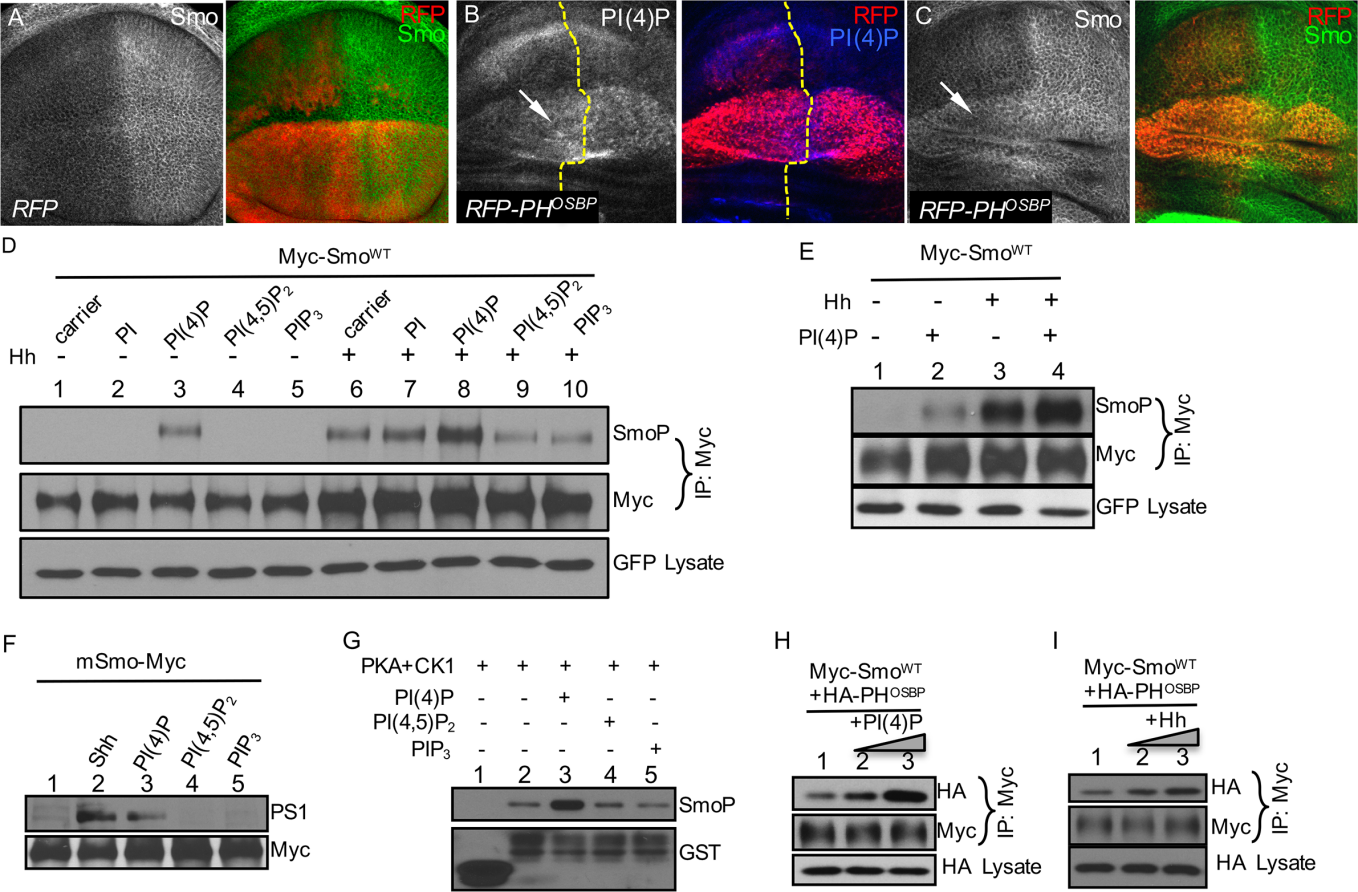
PI(4)P promotes Smo phosphorylation. (A) A wing disc expressing UAS-RFP by *MS1096*-Gal4 was stained for Smo. The expression of RFP has no effect on Smo accumulation. (B–C) Wing discs expressing RFP-PH^OSBP^ by *MS1096*-Gal4 were stained for PI(4)P and Smo. Arrows indicate accumulation of PI(4)P and Smo by PH^OSBP^ expression. (D) S2 cells were transfected with Myc-Smo^WT^ and GFP followed by the treatment with the indicated Ptdlns lipids, in combination with either Hh-conditioned medium or control medium. Cell extracts were immunoprecipitated with the anti-Myc antibody and analyzed by western blot using the anti-SmoP or anti-Myc antibodies. GFP served as a transfection and loading control. (E) S2 cells were transfected with Myc-Smo^WT^ and GFP, followed by the treatment of PI(4)P and Hh-conditioned medium. Immunoprecipitation of cell extracts with the anti-Myc antibody was followed by western blot analysis using the anti-SmoP and anti-Myc antibodies. Myc-Smo was normalized by the method described in Methods. GFP served as a transfection control. (F) NIH3T3 cells were transfected with mSmo-Myc and treated with Shh or the indicated PIPs, followed by western blot analysis using the anti-PS1 antibody to examine mSmo phosphorylation. (G) An in vitro kinase assay is shown using the purified GST-Smo^WT^ protein containing aa 656–755, the commercial PKA and CK1 kinases, and PI(4)P, PI(4,5)P_2_, PI(3,4,5)P_3_ (10 μM each). GST was used as a control. (H–I) S2 cells were transfected with Myc-Smo^WT^ and HA-PH^OSBP^, followed by treatment with PI(4)P (5 μM or 10 μM) or Hh-conditioned medium (30% or 60%). Immunoprecipitation of cell extracts with the anti-Myc antibody was followed by western blot with either the anti-Myc or anti-HA antibody to detect Smo-bound PH^OSBP^. Lysates blotted with the anti-HA antibody served as loading control.

The PH domain is a known phosphoinositide-binding module that is important for signal transduction by sensing alterations in the membrane lipid composition. To visualize PI(4)P pools in wing discs, we used an RFP-PH^OSBP^ reporter that contains two copies of the PH domain from the oxysterol binding protein (OSBP), which is known to specifically bind PI(4)P [45]. In wing discs, expression of RFP-PH^OSBP^ accumulated PI(4)P (Fig 2B) and Smo (Fig 2C), compared to the expression of RFP alone (Fig 2A). In cultured S2 cells, treatment with PI(4)P enhanced Smo activity, indicated by an elevated *ptc*-luc reporter activity (S3B Fig), thus prompting the question of whether PI(4)P regulates Smo phosphorylation, since phosphorylation promotes Smo activation. Indeed, we found that PI(4)P, but not other phospholipid forms, increased the levels of basal and Hh-induced Smo phosphorylation detected by a phospho-specific antibody (SmoP) [44] recognizing phosphorylation within the second PKA/CK1 cluster (Fig 2D). In addition, PI(4)P treatment induced Smo phosphorylation to a lesser extent compared to Hh treatment, and the combination of Hh and PI(4)P induced hyperphosphorylation of Smo (Fig 2E). Consistently, treatment with PI(4)P induced mSmo phosphorylation in cultured NIH3T3 cells, which was detected by a phospho-specific antibody (PS1) [30] recognizing mSmo phosphorylation at the first CK1/GRK cluster (Fig 2F). In an in vitro kinase assay using glutathione *S*-transferase (GST)-Smo fusion protein containing Smo amino acids 656–755, we found that Smo phosphorylation by PKA and CK1 kinases was enhanced by the addition of PI(4)P, but not PI(4,5)P_2_ or PIP_3_ (Fig 2G), suggesting that PI(4)P directly regulates the phosphorylation of Smo. In support of this notion, we found that Smo interacted with the PH domain from OSBP, and that this interaction was enhanced by the treatment with either PI(4)P or Hh (Fig 2H and 2I).

### PI(4)P Regulates Smo Phosphorylation through Direct Interaction with an Arginine Motif

**Fig 3:**
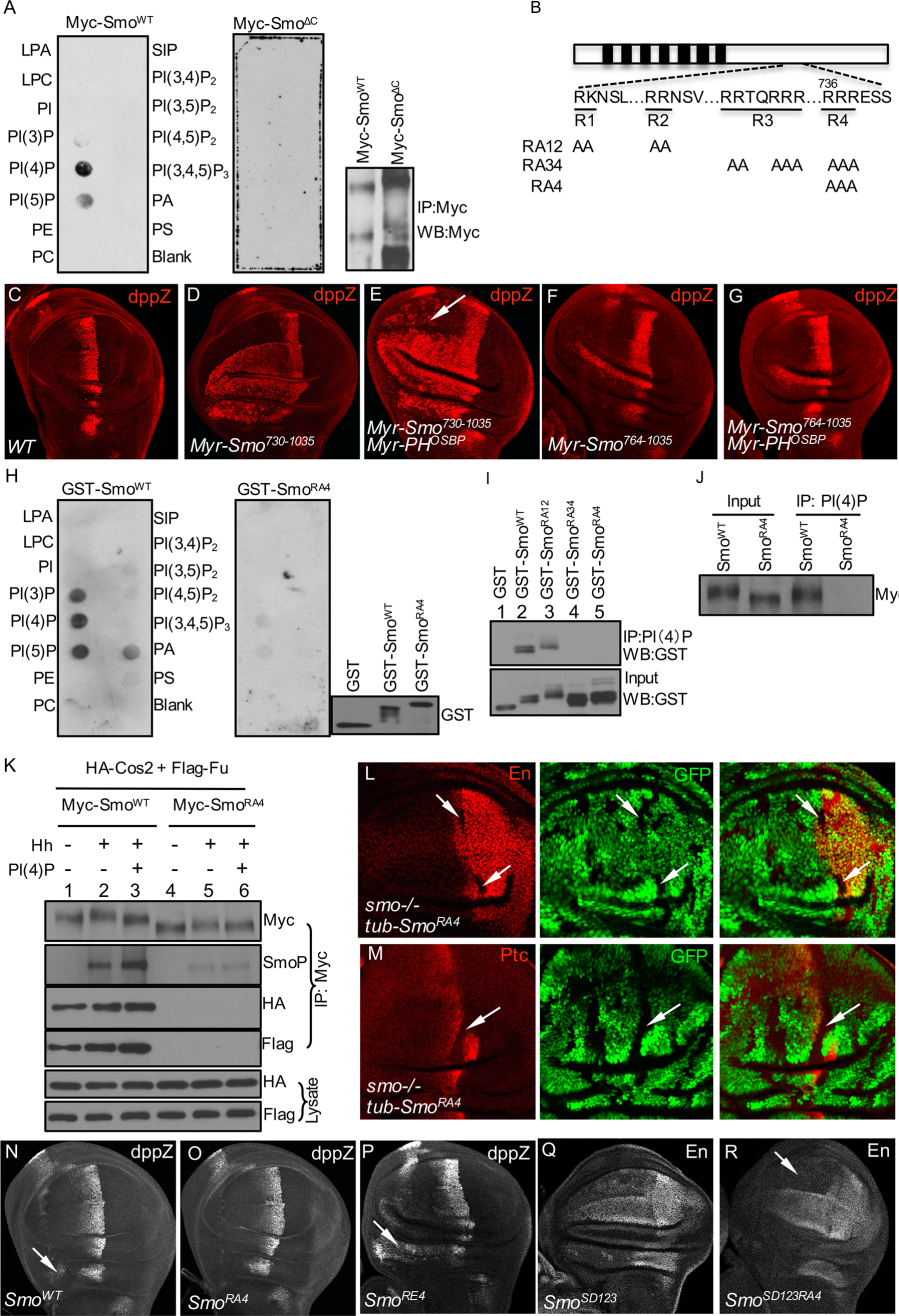
PI(4)P directly interacts with an arginine motif in Smo C-tail. (A) Smo interaction with phospholipids in a protein:lipid overlay assay. Lipid-dotted strips were incubated with Myc-Smo^WT^ (left) or Myc-Smo^ΔC^ (middle) purified from S2 cells, and bound Smo detected using the anti-Myc antibody. The PIP-Strip was dotted with 15 different lipids at 100 pmol per spot. LPA, lysophosphatidic acid; LPC, lysophosphatidylcholine; PI, phosphatidylinositol; PE, phosphatidylethanolamine; PC, phosphatidylcholine; S1P, sphinogosine-1-phosphate; PA, phosphatidic acid; PS, phosphatidylserine. Input Myc-Smo^WT^ and Myc-Smo^ΔC^ proteins are shown in the right panel. (B) Schematic drawing of full-length Smo with the sequences of the four arginine motifs shown underneath. Arginine residues were mutated as indicated. (C) A WT wing disc stained for *dpp*-lacZ expression. (D–G) Wing discs expressing Myr-Smo^730-1035^ (D–E) or Myr-Smo^764-1035^ (F–G), with or without the coexpression of Myr-PH^OSBP^ by *MS1096*-Gal4, were stained for *dpp*-lacZ expression. Arrow indicates the elevated *dpp*-lacZ expression. (H) The protein:lipid overlay assay was used as in (A) to examine GST-Smo^WT^ (left) or GST-Smo^RA4^ (middle) binding lipids on the lipid-dotted strip. Input GST or GST-Smo proteins are shown in the right panel. (I) The indicated GST or GST-Smo proteins were bacterially expressed, purified, and incubated with PI(4)P beads, followed by western blot analysis using the anti-GST antibody. Input for the incubation is shown in the lower panel. (J) S2 cells were transfected with either Myc-Smo^WT^ or Myc-Smo^RA4^ and immunoprecipitated with the anti-Myc antibody. Purified proteins were then incubated with PI(4)P beads, followed by western blot with the anti-Myc antibody to examine the bound Myc-tagged proteins. (K) S2 cells were transfected with the indicated constructs and treated with Hh-conditioned medium and/or PI(4)P, followed by immunoprecipitation with the anti-Myc antibody to detect Smo-bound Cos2 and Fu. Cell lysates were analyzed by western blot to examine the protein expression. (L–M) Wing discs containing *smo* mutant clones and expressing *tub*-Smo^RA4^ were immunostained to show En, Ptc, and GFP. Mutant clones are recognized by the lack of GFP expression and indicated by arrows. (N–R) Wing discs expressing the indicated Smo variants driven by *MS1096*-Gal4 were stained for *dpp*-lacZ or En. Arrow in (N) indicates the ectopic expression of *dpp*-lacZ induced by Smo^WT^. Arrow in (P) indicates the elevated *dpp*-lacZ expression compared to arrow in (N). Arrow in (R) indicates the reduction of En expression compared to (Q).

It is possible that PI(4)P directly interacts with Smo and facilitates Smo interaction with the PH domain of OSBP. To test this, we used a solid-phase lipid-binding assay and found that purified full-length Myc-Smo^WT^ strongly associated with PI(4)P, and weaker binding to PI(5)P was detected as well (Fig 3A, left column). Because the level of PI(5)P is much lower than that of PI(4)P in cells [46], PI(4)P is likely the primary lipid that binds Smo. We further found that Myc-Smo^ΔC^ (Smo lacking the C-tail) did not interact with PI(4)P (Fig 3A, right column), suggesting that the C-tail of Smo is required for binding to PI(4)P.

To identify the residues in the Smo C-tail interacting with PI(4)P, we used an in vivo approach to examine whether the activity of Smo is regulated by the PH domain expression. Membrane-tethered Smo C-terminal truncations by the myristoylation signal (Myr-SmoCT) possess the activity to induce ectopic *dpp*-lacZ expression [21]. We found that the membrane-tethered PH domain of OSBP (Myr-PH^OSBP^) increased the ectopic *dpp*-lacZ expression induced by Myr-Smo^730-1035^ but did not change *dpp*-lacZ expression induced by Myr-Smo^764-1035^ (compare Fig 3E to 3D and 3G to 3F), although Myr-PH^OSBP^ itself had no effect on *dpp*-lacZ expression in the wing. This suggests that PH^OSBP^ likely regulates Smo activity through aa 730–764. This domain of Smo contains one of the four positively charged arginine clusters, which are known to negatively regulate Smo activity by counteracting phosphorylation (Fig 3B) [20]. Surprisingly, using GST-Smo^656-755^ in the solid-phase lipid-binding assay, we found that Smo^WT^ strongly interacted with PI(4)P; however, an Arg to Ala mutation (GST-Smo^RA4^) abolished this interaction (Fig 3H), suggesting PI(4)P interacts with only the fourth arginine cluster. In support of this finding, mutation in the fourth arginine cluster (R4) was sufficient to block PI(4)P binding in a PI(4)P beads pull-down assay (Fig 3I). To further characterize R4, we generated the Arg to Ala mutation in Smo full-length (Myc-Smo^RA4^) and found that Myc-Smo^RA4^ lost both the ability to bind PI(4)P (Fig 3J) and the interaction with Cos2-Fu complex (Fig 3K). In addition, phosphorylation of Smo^RA4^ was no longer regulated by Hh and PI(4)P (Fig 3K). Smo^RA4^ also had no responsiveness to PI(4)P stimulation in the *ptc*-luc assay (S4E Fig). Using the fluorescence resonance energy transfer (FRET) assay to test C-terminal CFP/YFP dimerization [20,44], we found that Smo^RA4^ had a much lower FRET signal and was much less responsive to both Hh and PI(4)P stimulation compared to Smo^WT^ (S4F Fig). RA4 mutation did not cause protein misfolding because there was no difference between Smo^RA4^ and Smo^WT^ regarding the expression and subcellular distribution in S2 cells. Taken together, our findings suggest that Smo^RA4^ is inactive. Indeed, driven by the *tubulinα* promoter that expresses Smo at a level close to endogenous gene expression [17], the expression of Smo^RA4^ did not rescue *ptc* and *en* expression in *smo* mutant cells (Figs 3L, 3M and S4G). Furthermore, we found that overexpression of Smo^RA4^ by *MS1096*-Gal4 did not induce ectopic expression of *dpp*-lacZ, as noted with Smo^WT^ (Fig 3N and 3O), and that an R>A mutation in the constitutively active form of Smo (Smo^SD123RA4^) had lower *ptc*-luc activity compared to Smo^SD123^ (S4E Fig) and was unable to induce *en* expression in ventral A compartment cells (Fig 3R, compared to Fig 3Q). These data suggest that Smo activity is compromised by R>A mutation in the fourth arginine motif. In contrast, we found that R>E mutation (Smo^RE4^), which mimics negative charges caused by PI(4)P binding, elevated Smo activity to induce higher levels of *dpp*-lacZ expression in the wing disc (Fig 3P) and higher levels of *ptc*-luc activity, but had no responsiveness to PI(4)P stimulation (S4E Fig). Taken together, our findings suggest that the fourth arginine motif is required for Smo activation.

The binding position of PI(4)P in Smo is very critical, because fusion of the PH domain from OSBP to either the third intracellular loop (SmoL3PH) or the C-tail (SmoPH) retained PI(4)P with Smo (S4A Fig) but compromised Smo activity in the wing (S4C and S4D Fig, compared to S4B Fig) and resulted in loss of responsiveness to PI(4)P stimulation (S4E Fig). In comparison, fusion of CFP and YPF to the third intracellular loop and C-tail, respectively, did not change the activity of Smo [20]. Our findings suggest that PI(4)P binds Smo in a position-dependent manner.

### The PH Domain of Gprk2 Mediates Smo Regulation by PI(4)P

**Fig 4:**
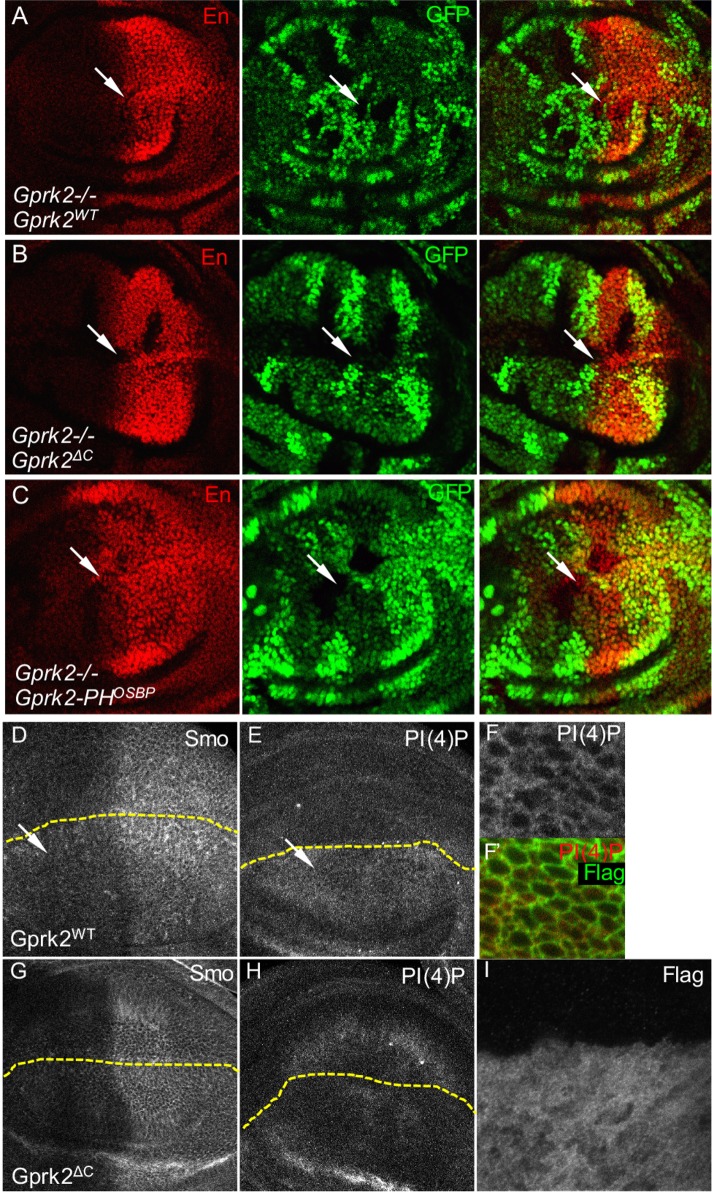
Kinase activity-independent role of Gprk2 by enriching PI(4)P in vivo. (A–C) Wing discs carrying *gprk2* clones and expressing Flag-Gprk2^WT^, Flag-Gprk2^ΔC^, or Flag-Gprk2-PH^OSBP^ by *MS1096*-Gal4 were immunostained for En and GFP. The *gprk2* mutant clones were marked by the lack of GFP expression. Arrows indicate the mutant clones near the A/P boundary. (D–F’) Wing discs expressing Flag-Gprk2^WT^ by the dorsal compartment-specific *ap*-Gal4 were treated with 100 μM PI(4)P (with 1:1 Carrier 3) and 0.1 μg/mL ecdysone in the M3 medium for 4 h and then immunostained for Smo, PI(4)P, and Flag. Dashed lines indicate the dorsal/ventral boundary marked by immunostaining with the anti-Flag antibody, which is not included here. Arrows indicate elevated levels of Smo and PI(4)P by the expression of Gprk2^WT^. (G–I) Wing discs expressing Flag-Gprk2^ΔC^ by *ap*-Gal4 were treated with PI(4)P as described above. Dashed lines indicate the dorsal/ventral boundaries.

**Fig 5:**
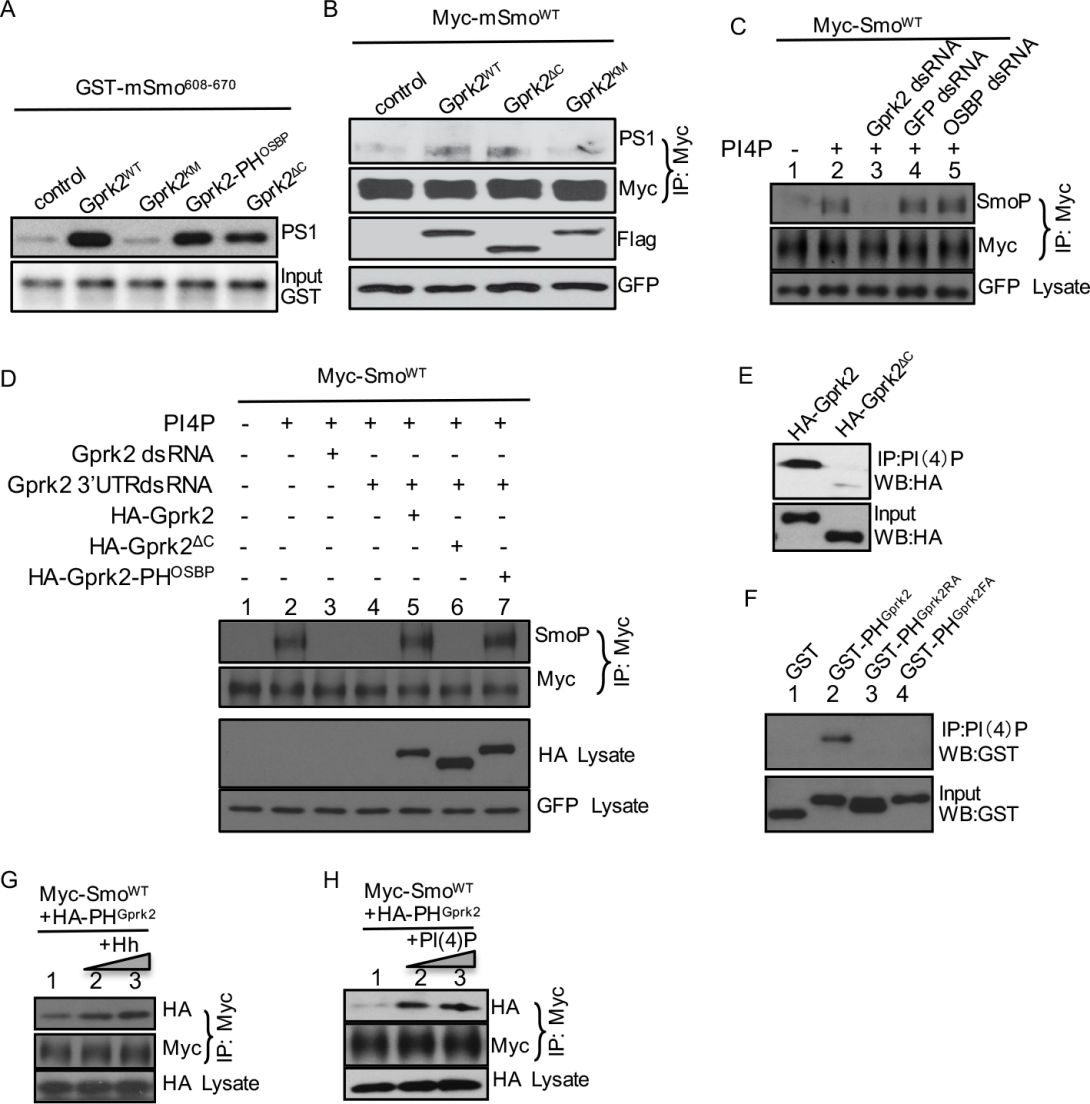
PH domain is required for Gprk2 to promote Smo phosphorylation. (A) An in vitro kinase assay using the GST-mSmo (mouse Smo aa 608–670) fusion protein and the indicated Flag-tagged Gprk2 proteins purified from S2 cells. (B) NIH3T3 cells were transfected with the indicated constructs and immunoprecipitated with the anti-Myc antibody, followed by western blot with the anti-PS1 antibody to detect mSmo phosphorylation. Of note, expressing Gprk2^ΔC^ induces mSmo phosphorylation at a level comparable to expressing Gprk2^WT^. (C) S2 cells were transfected with Myc-Smo^WT^ and GFP, treated with the indicated dsRNA in combination with either PI(4)P or carrier control, followed by immunoprecipitation using the anti-Myc antibody and western blot analysis using the anti-SmoP and anti-Myc antibodies. Myc-Smo normalization is described in S1 Methods. GFP served as transfection control. (D) S2 cells were transfected with Myc-Smo^WT^ and GFP, treated with either dsRNA targeting the coding region of Gprk2 or dsRNA targeting the 3′-UTR of Gprk2 in combination with transient transfection of HA-Gprk2, HA-Gprk2^ΔC^, or HA-Gprk2-PH^OSBP^. Cell extracts were immunoprecipitated with the anti-Myc antibody, followed by western blot analysis using the anti-SmoP and anti-Myc antibodies. Myc-Smo normalization is described in S1 Methods. GFP served as transfection control. (E) A PI(4)P beads pull-down assay examined binding of HA-Gprk2^WT^ or HA-Gprk2^ΔC^ purified from S2 cells transfected with each construct. (F) A PI(4)P beads pull-down assay examined bound GST-Gprk2 proteins purified from bacteria. (G–H) S2 cells were transfected with Myc-Smo^WT^ and HA-PH^Gprk2^ followed by treatment with Hh-conditioned medium (30% or 60%) or PI(4)P (5 μM or 10 μM). Immunoprecipitated cell extracts using the anti-Myc antibody was followed by western blot analysis with either the anti-Myc or anti-HA antibody to detect Smo bound PH^Gprk2^. Lysates blotted with the anti-HA antibody served as a transfection and loading control.

Considering that the PH domain of OSBP interacts with and activates Smo, we wondered whether a PI(4)P transport protein (PITP) facilitates the interaction between PI(4)P and Smo, since Smo itself does not contain a PH domain. We used RNAi lines from the VDRC to screen a total of 15 typical PH domain-containing PITPs in the fly genome for their ability to modulate Hh phenotypes; inactivation of these proteins by RNAi did not affect Smo accumulation in wing discs, although RNAi of some candidate PITPs modified the wing phenotype of *C765*-Smo^PKA12^ (S1 Table). Interestingly, all Gprks contain a PH domain in their C-terminus, and this domain contributes to agonist-dependent translocation by facilitating interaction with lipids and other membrane proteins [47,48]. We next investigated whether the C-terminus PH domain of Gprk2 was important for its role in Hh signal transduction. Wild-type Gprk2 fully rescued *en* expression in *gprk2* mutant cells (Fig 4A). However, deletion of the PH domain in the Gprk2 C-tail (Grpk2^ΔC^) abolished its ability to rescue *en* expression (Fig 4B), whereas replacing the PH domain in Gprk2 with the PH domain from OSBP (Gprk2-PH^OSBP^) restored this ability (Fig 4C). This is consistent with our previous finding that Gprk2KM, a kinase-dead form of Gprk2, has a kinase activity-independent role in regulating Smo [18]. These findings suggest that the PH domain is required for Gprk2 to fully function in transducing the Hh signal. In support of these results, Gprk2, Gprk2^ΔC^, and Gprk2-PH^OSBP^, but not Gprk2KM, were able to phosphorylate mSmo in vitro (Fig 5A), indicating that the removal or replacement of the PH domain does not affect the kinase activity. Thus, the function of the Gprk2 PH domain likely accounts for the kinase-independent role of Gprk2 in Smo regulation.

Because both Gprk2 transcription and Gprk2 protein expression are upregulated by Hh signaling, and Gprk2 is enriched at the A/P boundary [18,49], we hypothesize that, in addition to promoting the production of PI(4)P, Hh may regulate PI(4)P accumulation by enhancing the expression of Gprk2 as the endogenous carrier for PI(4)P. To examine the ability of Gprk2 to enrich PI(4)P in vivo, we knocked down Gprk2 in the wing disc and found that the levels of PI(4)P were decreased (S5A Fig). We also overexpressed Gprk2 or Gprk2^ΔC^ and found that the expression of Gprk2 elevated the levels of both Smo and PI(4)P (Fig 4D and 4E), whereas the expression of Gprk2^ΔC^ had no effect (Fig 4G and 4H). Similar to RFP-PH^OSBP^ (Fig 2B and 2C), overexpression of PH^Gprk2^ resulted in increased PI(4)P and Smo accumulation (S5B and S5C Fig). In addition, Gprk2 and PI(4)P were largely localized at the cell surface (Fig 4F and 4F′), whereas Gprk2^ΔC^ was cytosolic (Fig 4I). These results suggest that the PH domain of Gprk2 is required for the enrichment of PI(4)P in vivo by localizing Gprk2 at the cell surface.

To further characterize the kinase activity-independent role of Gprk2 in regulating Smo, we examined Gprk2-regulated Smo phosphorylation in cultured S2 cells. We found that RNAi targeting the coding region of Gprk2, but not OSBP, attenuated PI(4)P-induced Smo phosphorylation detected by the anti-SmoP antibody (Fig 5C). RNAi targeting the 3′-UTR region of Gprk2 consistently inhibited Smo phosphorylation (Fig 5D, lane 4, top panel). We found that the expression of HA-Gprk2 or HA-Gprk2-PH^OSBP^ rescued Smo phosphorylation inhibited by RNAi of Gprk2 3′-UTR but the expression of HA-Gprk2^ΔC^ did not (Fig 5D), suggesting that the PH domain is responsible for Gprk2 to promote Smo phosphorylation increased by PI(4)P. We also found that deletion of the PH domain decreased the Gprk2-PI(4)P interaction in the PI(4)P beads pull-down assay (Fig 5E). Moreover, the PH domain of Gprk2 (PH^Gprk2^) interacted directly with PI(4)P; mutation of arginine (PH^Gprk2RA^) or phenylalanine (PH^Gprk2FA^) abolished this interaction (Fig 5F). Finally, similar to the PH^OSBP^ interaction with Smo (Fig 2H and 2I), the PH^Gprk2^ interaction with Myc-Smo^WT^ was increased by Hh and PI(4)P treatments in cultured S2 cells (Fig 5G and 5H). Taken together with the observation that deletion of the PH domain does not alter the kinase activity of Gprk2 in vitro (Fig 5A) and in cultured NIH3T3 cells (Fig 5B), our findings suggest that the Gprk2 PH domain plays a positive role in mediating Smo regulation by PI(4)P.

### Hh Treatment Increases Smo-PI(4)P Interaction and Decreases Ptc-PI(4)P Interaction

**Fig 6:**
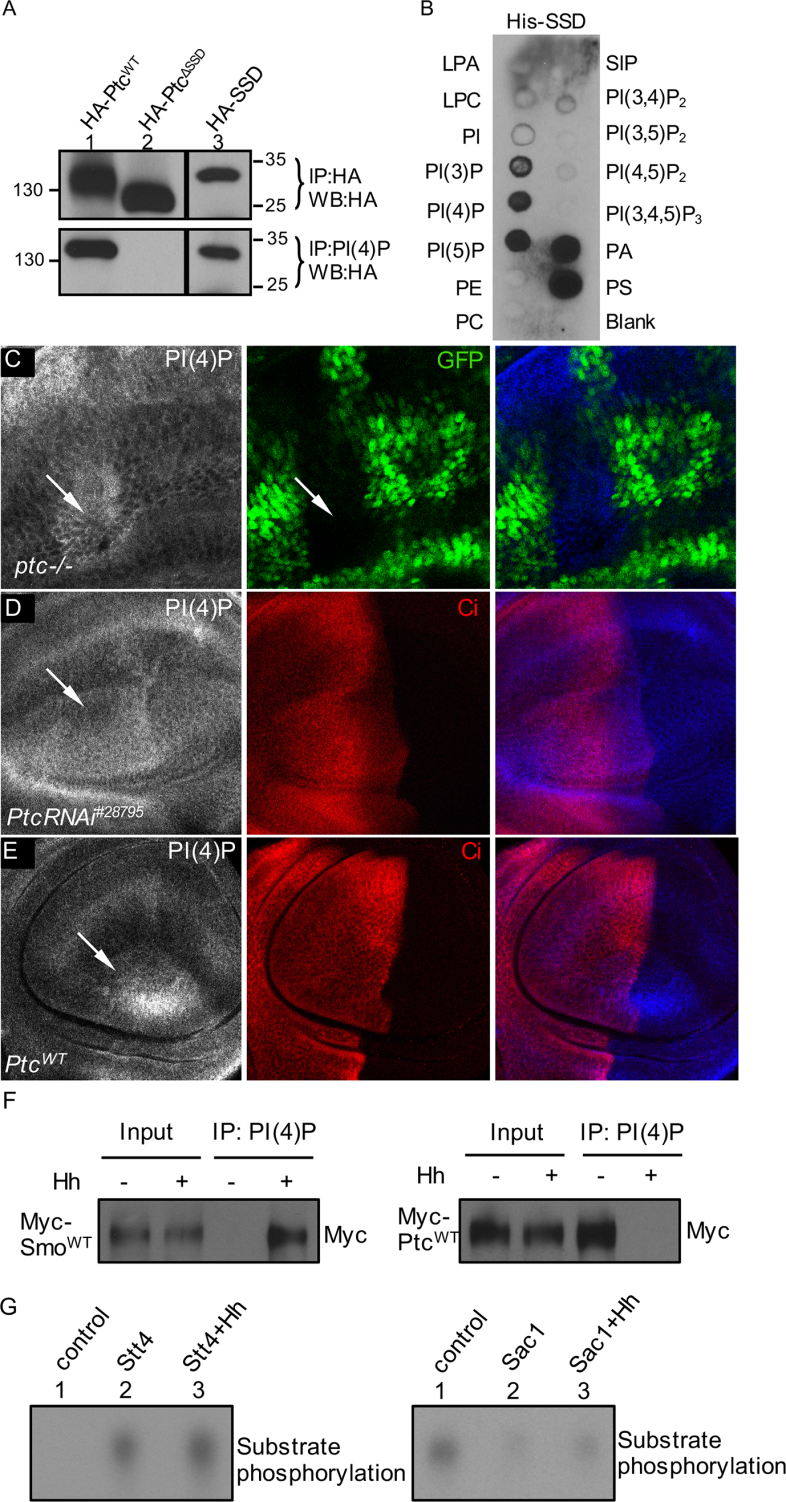
Ptc SSD domain interacts with PI(4)P. (A) S2 cells were transfected with the indicated HA-tagged Ptc constructs and then split in half. One half was used to detect Ptc protein by immunoprecipitation with the anti-HA antibody and western blot analysis using the anti-HA antibody. The second half was used to detect PI(4)P bound Ptc by lipid beads protein pull-down assay using PI(4)P beads and western blot using anti-HA antibody. The SSD domain is responsible for Ptc binding to PI(4)P. (B) Ptc binding in a protein:lipid overlay assay. Lipids dotted strips were incubated with bacterially expressed His-tagged SSD (His-SSD), and bound-SSD was detected with an anti-His antibody. (C) A wing disc bearing *ptc-/-* mutant clones was immunostained for PI(4)P and GFP. Arrows indicate the mutant clone marked by the lack of GFP expression. (D–E) Wing discs expressing either PtcRNAi^#28795^ or HA-Ptc^WT^ were stained for PI(4)P and Ci. Arrows indicate the accumulation of PI(4)P by RNAi and overexpression of Ptc. (F) S2 cells were transfected with either Myc-Smo^WT^ or Myc-Ptc^WT^, treated with Hh-conditioned medium or control medium, and immunoprecipitated with the anti-Myc antibody. Purified proteins were then incubated with PI(4)P beads followed by western blot with the anti-Myc antibody to examine the bound Myc-tagged proteins. Hh treatment increases the level of PI(4)P-bound Smo and decreases the level of PI(4)P-bound Ptc. Similarly, Hh treatment promotes Smo–PI(4)P interaction and inhibits Ptc–PI(4)P interaction in an independent assay using PI(4)P liposomes. (G) In vitro kinase assay (left) and in vitro phosphatase assay (right) were carried out to examine Stt4 and Sac1 enzymatic activity regulated by Hh. Stt4 and Sac1 were purified from S2 cells transfected with Flag-Stt4 or HA-Sac1 with the treatment of either Hh-conditioned medium or control medium. The substrates were chromatographed on oxalate-pretreated silica gel plate and visualized by autoradiography (see S1 Methods for details). The activity of Stt4 was enhanced by Hh treatment, indicated by the stronger signal (left panel, lane 3, compared to lane 2). In contrast, the activity of Sac1 was reduced by Hh treatment, indicated by the stronger signal (right panel, lane 3, compared to lane 2).

The finding that expression of the PH domain from OSBP accumulated PI(4)P (Fig 2B) prompted the notion that an endogenous protein may attract PI(4)P away from Smo in the absence of Hh. Ptc contains a sterol-sensing domain (SSD) and has structural similarity to the resistance, nodulation, division (RND) family of bacterial proton gradient-driven transmembrane molecular transporter [50]. SSD was first identified in proteins implicated in cholesterol metabolism but is now more broadly associated with vesicle trafficking. The Ptc SSD is essential for suppression of Smo activity [51], and mutations of SSD abrogate the Ptc-mediated repression of Smo, although these mutations do not compromise either binding or internalization of Hh [10,52]. It is possibe that the Ptc SSD controls the influx or the efflux of PI(4)P or attracts PI(4)P away from Smo. To test this hypothesis, we generated three vectors: HA-tagged wild-type full-length Ptc (HA-Ptc^WT^), HA-tagged Ptc lacking its SSD domain (HA-Ptc^ΔSSD^), and HA-tagged SSD domain (HA-SSD). We transfected S2 cells with these constructs and evaluated the ability of each to interact with PI(4)P. When expressed in S2 cells, all proteins were expressed at low levels, detected only after immunoprecipitation (Fig 6A, top panel). We found that HA-Ptc^WT^ and HA-SSD strongly bound PI(4)P, whereas HA-Ptc^ΔSSD^ did not bind (Fig 6A, lower panel).

To further determine whether the SSD domain from Ptc directly interacts with PI(4)P, we used the solid phase lipid-binding assay similar to that used for detecting Smo binding. We found that the SSD fragment protein purified from bacteria strongly associated with PI(4)P, but not with PIP_2_ or PIP_3_ phospholipids (Fig 6B), suggesting that the interaction between SSD and PI(4)P in the lipid beads protein pull-down assay is direct. The SSD association with PI(3)P or PI(5)P (Fig 6B) suggests that the expression of a single SSD domain may lose specificity for interaction, or, alternatively, that such interaction may also promote Ptc regulation of PI(3)P and PI(5)P. We also found a very strong interaction between SSD and phosphatidic acid (PA) or phosphatidylserine (PS) (Fig 6B); these may be nonspecific, as PA and PS binding to short protein fragments has often been considered questionable [53]. Our findings in cultured cells led us to examine the correlation of Ptc and PI(4)P in the wing. We found that mutation of *ptc* or knockdown of Ptc by RNAi increased PI(4)P levels in the wing disc (Fig 6C and 6D), similar to the observation that Ptc inactivation elevates PI(4)P in the salivary gland [42]. These indicate that the activation of Hh signaling by the inactivation of Ptc elevated the production of PI(4)P. Moreover, we found that the overexpression of Ptc^WT^ also increased the level of PI(4)P (Fig 6E), which is likely due to the ability of Ptc to accumulate PI(4)P. In support of these findings, Ptc^ΔSSD^ overexpression had no effect on regulating the accumulation of Smo, Ci, and PI(4)P in wing discs.

In addition to promoting the production of PI(4)P, Hh may also regulate the pools of PI(4)P between Smo and Ptc. To test this hypothesis, we purified Smo and Ptc proteins from S2 cells treated with Hh-conditioned medium or control medium and accessed the protein interaction with PI(4)P. As shown in Fig 6E, the level of PI(4)P-bound Smo was increased by the treatment of Hh (Fig 6F, left panel). In contrast, the level of PI(4)P-bound Ptc was decreased by Hh treatment (Fig 6F, right panel). These data indicate that Hh treatment releases PI(4)P from Ptc, suggesting an additional layer of regulation beyond Hh promoting PI(4)P production.

It would be interesting to understand how Hh regulates PI(4)P. However, we found that Hh treatment did not significantly change the mRNA levels of Stt4 and Sac1 (S6A Fig). In addition, Hh did not change the protein levels of the overexpressed Stt4 and Sac1 in P compartment cells of the wing disc (S6B and S6C Fig). Hh also did not regulate the accessibility of the Stt4/Sac1 to Smo or Ptc in an immunoprecipitation assay with S2 cells. To examine whether Hh regulates the activity of Stt4 or Sac1, or both, we carried out in vitro kinase/phosphatase assays using purified Stt4 and Sac1 combined with PI substrate. We found that the phosphorylation of PI was enhanced when using Stt4 from cells treated with Hh (Fig 6G, lane 3, compared to lane 2, left panel). In addition, Sac1 from cells treated with Hh had less activity to dephosphorylate the constitutive PI phosphorylation (Fig 6G, lane 3, compared to lane 2, right panel). These data suggest that Hh elevates the activity of Stt4 and inhibits the activity of Sac1.

### PI(4)P Promotes mSmo Activation and Localization in the Cilium

**Fig 7:**
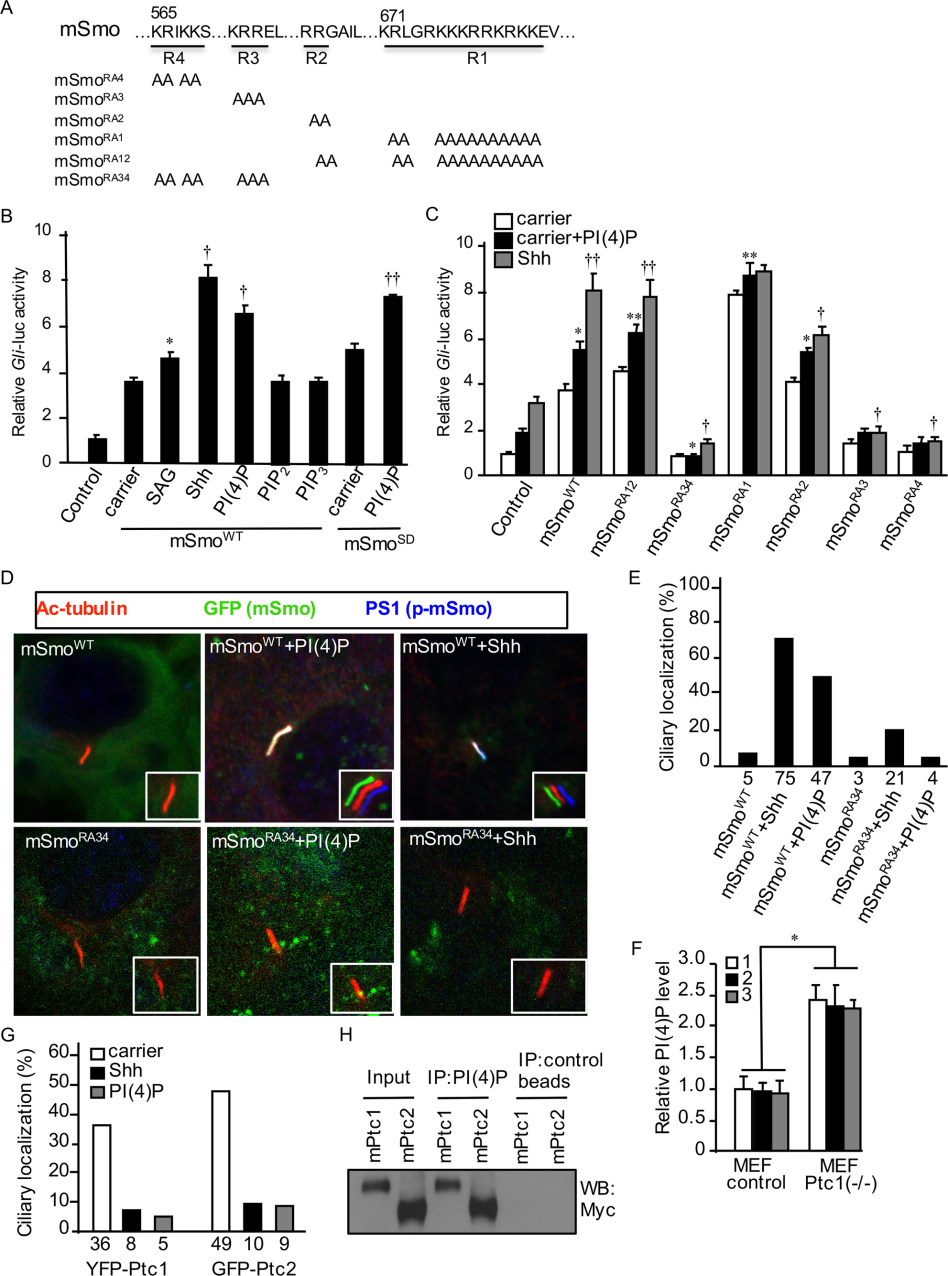
PI(4)P regulates the ciliary accumulation of mSmo, Ptc1, and Ptc2. (A) Schematic drawing of mSmo mutations in the four arginine motifs. (B) *Gli*-luc reporter assay in NIH3T3 cells transfected with blank vector (control), mSmo^WT^, or mSmo^SD^ and treated with SAG, Shh, or individual lipid. Carrier 3 served as the treatment control. * *p* < 0.01 versus carrier. † *p* < 0.001 versus carrier. †† *p* < 0.01 versus carrier in the mSmo^SD^ group. (C) *Gli*-luc assay in NIH3T3 cells transfected with blank vector (control) or each mSmo construct and treated with either PI(4)P or Shh. * *p* < 0.01 versus control plus PI(4)P. ** *p* < 0.001 versus control plus PI(4)P. † *p* < 0.01 versus control plus Shh. †† *p* < 0.001 versus control plus Shh. Note, the overexpressed mSmo induced higher levels of Hh pathway activation, compared to Shh treatment, which might be due to the Shh peptides used or the high level expression of the transfected mSmo. (D) NIH3T3 transfected with EGFP-tagged mSmo^WT^ or mSmo^RA34^ and treated with Shh or PI(4)P were immunostained to show the expression of Acetylated (Ac)-tubulin (red; primary cilium), GFP (green; mSmo), and PS1 (blue; phosphorylated mSmo). Images in the inserts are enlarged views with shifted overlays to show the ciliary localization of mSmo. About 100 ciliated cells were counted for each set. (E) Quantification of ciliary localization of infected mSmo^WT^ and mSmo^RA34^ as indicated by the percentage of GFP^+^ cilia shown in (D). (F) ELISA assay was carried out to examine the levels of PI(4)P in either control MEFs or *ptc1-/-* MEFs. Bars indicate three independent repeats. * *p* < 0.001. (G) NIH3T3 cells transfected with YFP-Ptc1 or GFP-Ptc2 and treated with Shh or PI(4)P were immunostained to examine the ciliary localization of Ptc1 and Ptc2 (see S7B and S7C Fig for example images). About 100 ciliated cells were counted for each set. The relative small numbers of Ptc1 and Ptc2 in the cilia are likely due to transient transfection and the expression levels of the protein; however, statistical analysis data was collected from the same sets of cells. (H) Myc-Ptc1 or Myc-Ptc2 was transfected into NIH3T3 cell, purified by immunoprecipitation with the anti-Myc antibody, and incubated with PI(4)P beads or control beads followed by western blot with the anti-Myc antibody to examine the bound Myc-tagged proteins. The underlying data of panels B–C and E–G can be found in S1 Data.

Next, we wondered whether PI(4)P plays a role in regulating mSmo, because PI(4)P induces mSmo phosphorylation (Fig 2F). We first tested different phospholipids for their effects in activating mSmo and found that, similar to Shh, PI(4)P treatment elevated mSmo activity as monitored by a *Gli*-luc reporter (Fig 7B). In contrast, PIP_2_ and PIP_3_ treatment had no effect on mSmo activity. In addition, the activity of the constitutively active form of mSmo (mSmo^SD^), which mimics mSmo phosphorylation by GRK2 and CK1 [30], was further increased by PI(4)P (Fig 7B). Consistently, *Drosophila* Smo^SD123^ activity was increased by PI(4)P (S4E Fig).

Similar to *Drosophila* Smo, mSmo contains arginine clusters in its C-tail (Fig 7A) [20]. We next examined whether the arginine motif(s) were responsible for regulation of mSmo by PI(4)P. As shown in Fig 7C, PI(4)P treatment increased *Gli*-luc reporter activity, although to a lesser extent compared to Shh treatment in the control group of NIH3T3 cells. PI(4)P and Shh treatment consistently increased *Gli*-luc activity when cells were transfected with mSmo^WT^ (Fig 7C). However, R>A mutations in R3 and R4 arginine clusters (mSmo^RA3^ and mSmo^RA4^, respectively) attenuated the increased activity noted with PI(4)P, and mutations in both R3 and R4 (mSmo^RA34^) completely blocked the effect of PI(4)P on mSmo activation (Fig 7C). These data suggest that R3 and R4 are responsible for the regulation of mSmo by PI(4)P.

Phosphorylation promotes ciliary accumulation of mSmo, which correlates with pathway activation [30], but the molecular mechanisms that control Smo ciliary accumulation are poorly understood. mSmo^WT^ was found in about 5% of cilia, and Shh treatment increased Smo^WT^ accumulation in 75% of cilia (Fig 7D and 7E) [30]. Treatment with PI(4)P induced mSmo^WT^ accumulation in 47% of cilia (Fig 7D and 7E), which was correlated with changes in mSmo^WT^ phosphorylation (Fig 2F) and activity (Fig 7B) induced by PI(4)P. In contrast, mSmo^RA34^ had no response to PI(4)P treatment (4% of ciliary accumulation by PI(4)P treatment) (Fig 7D and 7E), although it had a low response to Shh stimulation (from 3% to 21% of ciliary localization). Consistent with these findings, mSmo^RA34^ had much lower activity and much less responsiveness to Shh stimulation in a previous study [20]. Our findings suggest that R3 and R4 clusters are responsible for PI(4)P-associated binding and activation of mSmo.

**Fig 8:**
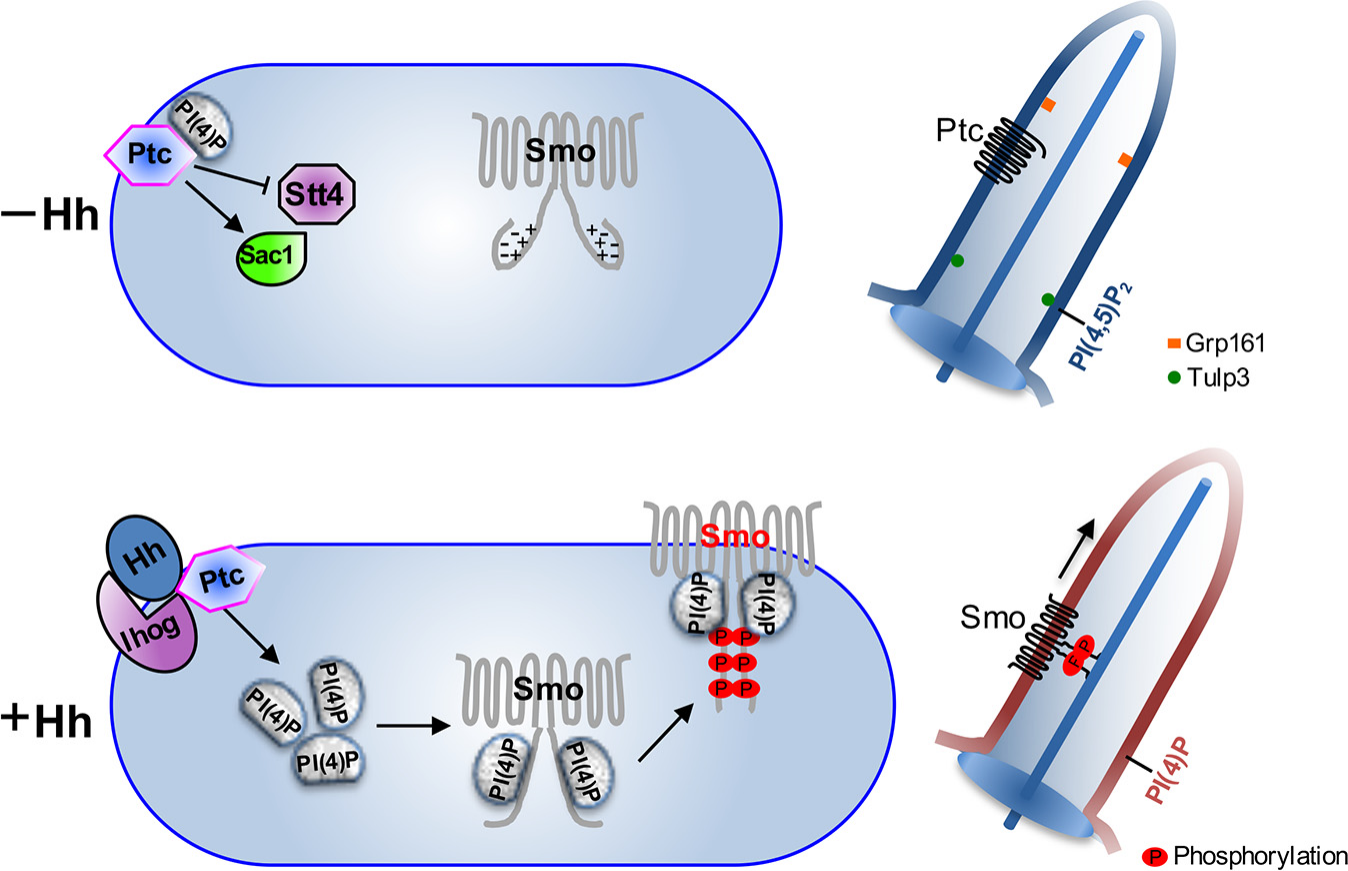
PI(4)P regulates the phosphorylation and ciliary accumulation of Smo. Models are based on this study and studies from recent publications. In the absence of Hh, Stt4 has low activity, whereas Sac1 has high activity, resulting in low levels of PI(4)P that interact with Ptc. In the vertebrate cilium, Ptc1/2 and Grp161 localize in the cilium to inhibit Hh signaling. PI(4,5)P_2_ accumulates in the cilium, but not PI(4)P. Upon Hh stimulation, PI(4)P production is increased and PI(4)P is also released from Ptc, allowing PI(4)P to bind and activate Smo by promoting Smo phosphorylation and dimerization. In the vertebrate cilium, PI(4)P levels are elevated by Hh, which promotes Smo phosphorylation and ciliary accumulation. PI(4)P elevated by Hh inhibits the ciliary localization of Ptc1/2.

To determine whether Hh regulates the production of PI(4)P in vertebrate systems, using the ELISA assay combined with the anti-PI(4)P antibody, we examined the levels of PI(4)P in *ptc1* mutant mouse embryonic fibroblasts (MEFs) and found that, compared to control MEFs, *ptc1* MEFs had significantly increased PI(4)P (Fig 7F). Consistently, Shh treatment increased, whereas Smo inhibitor decreased, the levels of PI(4)P (S7A Fig), indicating that Hh signaling activity promotes PI(4)P production in cultured cells. We further investigated the ciliary localization of Ptc1 and Ptc2 and found that the ciliary localization of both Ptc1 and Ptc2 was decreased by Shh treatment or PI(4)P treatment (Fig 7G), which was consistent with the previous study that Hh inhibits the ciliary localization of Ptc1 [54]. Similar to *Drosophila* Ptc interaction with PI(4)P, we found that both Ptc1 and Ptc2 interacted with PI(4)P in the lipid beads protein pull-down assay (Fig 7H). These observations indicate a consistent regulation of Smo and Ptc by PI(4)P in *Drosophila* and mammalian systems. To incorporate the findings in this study and the findings published recently [32,33,42], we proposed a model in which Smo phosphorylation and ciliary accumulation is regulated by PI(4)P (Fig 8).

## Discussion

Hh signal transduction has been widely studied; however, the longstanding questions of how Ptc inhibits Smo activity and how Hh promotes Smo phosphorylation and activation remain. These issues constitute a primary focus of this study. A genetic analysis indicates that inactivation of Stt4 downregulates Smo accumulation, whereas knockdown of Sac1 by RNAi elevates Smo levels [42], suggesting the involvement of phospholipids in Hh signal transduction. Nevertheless, the mechanisms by which phospholipids regulate Hh signaling remain unknown. In this study, we identified and characterized a direct role for PI(4)P in binding Smo and promoting Smo phosphorylation and activation. The mechanism by which PI(4)P interacts with arginine motifs in Smo is likely conserved between *Drosophila* Smo and mouse Smo, as such motifs have been mapped in both species.

### Kinase Activity-Independent Role of Gprk2 in Hh Signaling

To explore the possible involvement of a PITP protein to facilitate the interaction between Smo and PI(4)P, we unexpectedly found that the PH domain of Gprk2 is responsible for the accumulation of PI(4)P that activates Smo. It has been shown that Gprk2 is positively involved in Hh signaling by directly phosphorylating Smo C-tail [18]. In addition, Gprk2 forms a dimer/oligomer and binds Smo C-tail in a kinase activity-independent manner to promote Smo dimerization and activation [18]. However, how Gprk2 promotes Smo dimerization and activation is unclear. In this study, we found that the function for Grpk2 PH domain to activate Smo is independent of its kinase activity (Fig 5A and 5B) and that the PH domain of Gprk2 is responsible for enriching PI(4)P that promotes Smo phosphorylation and dimerization. There are instances in which the binding of lipids to the PH domain promotes dimerization of the protein [55,56], raising the possibility that PI(4)P interaction with the PH domain of Gprk2 promotes its dimerization. Taken together, our findings suggest that the function of PH domain in Gprk2 accounts, at least in part, for the kinase activity-independent role of Gprk2 in Hh signaling, a deeper mechanism for Smo activation by Gprk2.

### The Release of PI(4)P from Ptc in Response to Hh Stimulation

In the absence of Hh, Ptc inhibits Smo activity by promoting Smo endocytosis and turnover in intracellular compartments [9]. Ptc likely inhibits Smo catalytically [10], because substochiometric levels of Ptc are able to repress Smo activation [10,11]. Here, we found that Hh promotes the activity of Stt4 and inhibits the activity of Sac1 (Fig 6G), which, at least in part, explains the catalytic regulation.

A previous study proposed a model in which Ptc represses Smo by regulating lipid trafficking; Ptc recruits lipoproteins to endosomes, changing their lipid composition, in order to regulate Smo degradation [57], but the class of lipids remains unidentified. In the presence of Hh, Smo is phosphorylated and accumulates at the cell surface, resulting in protein activation [1,8]. However, it is unknown how Ptc inhibition on Smo is relieved by Hh stimulation. The ability of the Ptc SSD domain to interact with PI(4)P (Fig 6A and 6B) raises the possibility that Ptc may control the pool of phospholipids regulating the accessibility of Smo to PI(4)P. Our finding that Hh treatment decreased the interaction between Ptc and PI(4)P (Fig 6F) suggests the possibility that binding of Hh to Ptc results in a conformational change in Ptc and releases phospholipids. Thus, this study uncovers an additional layer of regulation by indicating the release of PI(4)P from Ptc, which may account for the optimal regulation of Smo.

### The Location of Smo Motif Responsible for Binding PI(4)P Is Critical for Changing Smo Conformation

The structure of the Smo N-terminal, including the extracellular cysteine rich domain (CRD), has been characterized [58,59]. Unlike other GPCRs, no ligand-binding function has been identified. It has been shown that Smo-mediated signal transduction is sensitive to sterols and oxysterol derivatives of cholesterol [60–62]. However, unlike vertebrate Smo, *Drosophila* Smo CRD does not interact with oxysterols [63]. In this study, we found that phospholipids activate both vertebrate and *Drosophila* Smo through binding to the arginine motif in the Smo C-terminus, although the C-tails have sequence divergence among species. Using the protein:lipid overlay assay, we found that PI(4)P directly binds Smo (Fig 3A) and that mutation in the R4 arginine motif abolishes this direct interaction (Fig 3H–3J). Importantly, R>A mutation abolished the activity of Smo (Figs 3L, 3M and S4G). It is likely that binding of PI(4)P to the arginine motif changes Smo conformation, thus allowing kinases to phosphorylate and activate Smo. In support of this notion, PI(4)P binding to specific arginine residues in specific locations is critical for Smo conformational change, because fusion of the PH domain to either the third intracellular loop or the C-terminus attracts PI(4)P to different locations in Smo, thus blocking Smo activation by PI(4)P (S4 Fig).

In this study, we focused on the regulation of Smo by PI(4)P and found that Hh regulates the accessibility of Smo to PI(4)P, evidenced by the Hh-promoted interaction between Smo and PH domain from either OSBP or Gprk2 (Figs 2I and 5E) and by the Hh-enhanced interaction between Smo and PI(4)P (Fig 6F). Binding of PI(4)P likely changes the conformation of Smo, leading to Smo phosphorylation by kinases. It should be noted that phosphomimetic Smo mutations (Smo^SD123^ and mSmo^SD^) are still regulated by PI(4)P (Figs 7B and S4E), suggesting that PI(4)P either promotes phosphorylation at other residues or has additional role(s) in activating Smo.

### Mechanisms for Hh to Promote PI(4)P Production

Hh signaling activity promoted the production of PI(4)P that was detected by the mass-spec assay (Fig 1C), which was a very sensitive approach. However, in a previous study, the overexpression of full-length wild-type Ci did not elevate the accumulation of PI(4)P in wing discs [42]. It is possible that low levels of Hh signaling activity induced by the expression of wild-type Ci are unable to induce detectable changes in PI(4)P accumulation in wing discs. The disc immunostaining with the anti-PI(4)P antibody might not be as sensitive as the mass-spec method. In support, by expressing Smo^SD123^ or the constitutively active Ci^-3P^, in which three PKA sites in the phosphorylation clusters were mutated to block Ci processing [64], we found that PI(4)P was accumulated in wing discs (S2A Fig), likely due to the high levels of Hh signaling activity induced by the active forms of Smo and Ci.

## Experimental Procedures

### Constructs, Mutants, and Transgenes

Generation of the Myc-Smo^WT^, Myc-Smo^ΔC^, Myr-Smo^730-1035^, and Myr-Smo^764-1035^ constructs and transgenes was previously described [21]. Myc-SmoPH and Myc-SmoL3PH were constructed by fusing one copy of the OSBP PH domain (aa 17–123) to the Smo C-terminus and two copies of the OSBP PH domain after aa 451 of Smo intracellular loop 3, respectively. Generation of the Myc-Smo^SD123^ and Myc-Smo^RA1234^ was previously described [15,20]. Myc-Smo^SD123RA4^ was generated by the combination of SD123 and RA4. Myc-tagged Smo^RA1^, Smo^RA2^, Smo^RA3^, Smo^RA4^, Smo^RA12^, and Smo^RA34^ were generated by PCR. To construct *tub*-Smo^RA4^, the previously described *tubulinα* promoter [17] was inserted upstream to the Smo^RA4^ sequence. Transgenic lines were generated using the VK5 attP locus to ensure Smo protein expression at the same levels without positional effects. Genotypes for examining the activity of Smo transgene in *smo* clones include: *yw hsp-flp*/+ or Y, *smo3 FRT40*/*hs-GFP FRT40*, and *tub-Smo* ^*RA4*^/+. RFP-PH^OSBP^ and RFP-PH^Gprk2^ were constructed by an in-frame fusion of two copies of the PH domain from OSBP or Gprk2 at the RFP C-terminus in the attB-UAST backbone [41]. RFP-PH^OSBP^ and RFP-PH^Gprk2^ transgenes were generated by insertion at the VK5 attP locus. As a control, the UAS-RFP transgenic line was generated with the same approach. Flag-Gprk2^WT^ and Flag-Gprk2^KM^ constructs and transgenes have been described [18]. Flag-tagged Gprk2^ΔC^ (containing Gprk2 aa 1–666) and Gprk2-PH^OSBP^ (aa 667–714 replaced by the PH domain of OSBP) were inserted in-frame in the Flag-UAST vector, and their transgenic lines were generated using standard P-element-mediated transformation. Multiple independent transgenes were generated, and those on the second chromosomes were used for rescue experiments. *Gprk2* mutant clones were generated with *yw 122* and *FRT82 Gprk2/FRT82 hs-Myc-GFP*. *ptc* mutant clones were generated with *yw hsp-flp/+* or Y and *ptc* ^*[wII]*^ *FRT42D/hs-GFP FRT42D*. Inp54p (from *Saccharomyces cerevisiae*, Addgene 20155) and IpgD (from *Shigella flexneri*, a gift from Dr. Frederique Gaits-Iacovoni) were subcloned into HA-UAST backbones. A phosphatase-dead version of Inp54p with a D281A mutation (Inp54p^D281A^) was generated by site-directed mutagenesis. GST-Smo^WT^ containing aa 656–755 has been described [15]. GST-tagged Smo^RA12^, Smo^RA34^, and Smo^RA4^ were generated by PCR on the backbone of GST-Smo^WT^. GST-PH^Gprk2^, GST-PH^Gprk2RA^, and GST-PH^Gprk2FA^ were constructed by the same approach. HA-Ptc^WT^, HA-Ptc^ΔSSD^, and HA-SSD were generated by PCR with Ptc or its fragments inserted into HA-UAST backbones, and transgenic lines were generated using the VK5 attP locus. His-SSD was generated by in-frame fusion of Ptc SSD domain (aa421-589) to pET30a backbone. Myc-tagged mSmo^WT^, mSmo^RA^ mutants, and mSmo^SD^ (previous mSmo^SD0-5^) have been described [20,30]. GFP-tagged mSmo^WT^ and mSmo^RA34^ were generated by subcloning each cDNA into pEGFP-N1 backbone. Flag-tagged Stt4 (Flag-Stt4) was generated by combining seven RT-PCR fragments with unique cloning sites into the SK^+^ backbone and, finally, subcloning into Flag-UAST backbone. HA-tagged Sac1 (HA-Sac1) was constructed by inserting the RT-PCR fragment into the HA-UAST backbone. pGE-mPtch1-Myc and pGE-mPtch2-Myc were generated by subclone of the open reading frame from pEGFP-mPtch1 and pEGFP-mPtch2 (Gift from Dr. Chi-Chung Hui), respectively. *MS1096*-Gal4, *ap-*Gal4, *C765*-Gal4, and *Gprk2* deletion mutants have been described [17,18]. Stt4 RNAi lines (v15993 and v105614), Sac1 RNAi lines (v44376, v37216, and #56013), Ptc RNAi line (#28795), Hh RNAi line (v1402), GFP-Sac1 (#57356 and #57357), and *prd*-Gal4 (#1947) were obtained from VDRC or Bloomington Stock Center (BSC). Generation of Gprk2 RNAi lines have been described [18]. The RNAi lines that targeted each PITP were obtained from either VDRC or BSC (S1 Table).

### Phospholipid Quantification by Mass Spectrometry and ELISA Assays

PtdIns lipid extraction and quantification by mass spectrometry were carried out as previously described [43]. Briefly, 3 × 10^7^ S2 cells (or 4 × 10^6^ NIH3T3 cells) from a 100 mm dish were harvested and washed once with ice-cold PBS and suspended in 340 μL H_2_O and 1500 μL quench mix with 25 ng of 17:0–20:4 PI(4)P (Avanti Polar Lipids, Inc.) in 10 μL methanol as the internal standard. Lipids were extracted with 1450 μL CHCl_3_ and 340 μL 2 M HCl and subsequently derivatized with trimethylsilyl diazomethane (Sigma), as previously described [43]. After washing and drying under a stream of nitrogen at room temperature, samples were dissolved in 200 μL methanol. We applied 10 μl of each sample to LC-MS/MS analysis using a Shimadzu LC-20 HPLC and TSQ Vantage triple quadrupole mass spectrometer (ThermoFisher). A Jupiter 5μ C4 300A (50 × 1.0 mm) column (Phenomenex) was used with the multiple reaction monitoring (MRM) transitions described [43]. PIP concentrations were calculated from MRM peak areas and the internal standard and were subsequently normalized to cell number.

For PI(4)P quantification by ELISA assay, similar PtdIns lipid extraction procedures were used without adding the PI(4)P internal control. After washing and drying with nitrogen stream, lipid extracts were dissolved in ethanol and loaded into a microplate, dried under a vacuum, and incubated with 2% BSA in PBS at room temperature for 30 min. Mouse anti-PI(4)P or anti-PI(4,5)P_2_ monoclonal antibodies (Echelon Biosciences) were added for 1 h, followed by goat anti-Mouse IgG-HRP (Jackson ImmunoResearch) for 30 min, with 3 PBS washes after each inculation. Finally, chemiluminescence substrate (SuperSignal West Pico, Pierce) was added to the microplate, and luminescence intensity was determined by a luminometer.

### Protein–Lipid Overlay and PtdIns Lipid Pull-Down Assays

GST-Smo fusion proteins expressed in bacteria were pooled by GST beads (GE Healthcare), then eluted with elution buffer (10mM Glutathione pH 8.0 in 50 mM Tris) at 4°C overnight. Myc-tagged Smo proteins expressed in S2 cells were immunoprecipitated with anti-Myc antibody combined with beads of protein A ultralink resin, followed by two sequential elutions with Myc peptide (Roche, 100mM KCl, 20% glycerol, 20 mM HEPES KOH, pH 7.9, 0.2 mM EDTA, 0.1% NP-40, 5 mM DTT, and 0.5 mM PMSF). The eluted purified GST-fusion proteins and Myc-tagged Smo proteins were concentrated by the Centrifugal filter units (Millipore) and incubated with lipid-dotted strips according to manufacturer’s instruction (Echelon Biosciences), followed by western blot with the anti-GST (Santa Cruz), anti-Myc (Santa Cruz), or anti-His (Millipore) antibodies.

For the PI(4)P beads pull-down experiments, HA-tagged Gprk2 proteins were expressed in S2 cells, immunoprecipitated with mouse anti-HA antibody (F7, Santa Cruz), eluted with HA peptide (Sigma, in 500 mM NaCl), and concentrated by the Centrifugal filter units (Millipore). GST-PH^Gprk2^, GST-PH^Gprk2RA^, and GST-PH^Gprk2FA^ proteins were expressed in bacteria and purified using the protocol employed for GST-Smo purification. The purified and concentrated GST-fusion proteins or epitope-tagged proteins were incubated with PI(4)P beads (Echelon Biosciences) with wash/binding buffer (10 mM HEPES, pH7.4; 0.25% NP-40; 150 mM NaCl), and subjected to western blot to detect PI(4)P bound proteins.

### Immunostaining of Wing Imaginal Disc, *Drosophila* Embryo, and NIH3T3 Cell Cilia

Wing discs from third instar larvae were dissected in PBS and then fixed with 4% formaldehyde in PBS for 20 min. After permeabilization with 1% PBST, discs were incubated with primary antibodies for 3 h and appropriate secondary antibodies for 1 h, and washed three times with PBST after each incubation. Affinity-purified secondary antibodies (Jackson ImmunoResearch) for multiple labeling were used. It was a challenge for disc staining with the mouse anti-PI(4)P antibody. We have adopted/modified a critical method for PI(4)P immunostaining from previous publications [46,65]. Discs were fixed in 4% formaldehyde in PBS and permeabilized in 1 M sucrose by freezing at -80°C for 1 h followed by thawing at room temperature. Then discs were washed with PBS and incubated with 50 mM NH_4_Cl for 15 min, followed by incubation with the anti-PI(4)P antibody at 4°C overnight. For *Drosophila* embryo primary antibody staining, stage 11 fly embryos with specific genotypes were dechorionated, fixed with Heptane solution, and immunostained with similar procedures. To examine mSmo ciliary localization, NIH3T3 cells were transfected with mSmo-GFP variants, treated with Shh or PI(4)P, and immunostained for mSmo localization in the cilium. Primary antibodies in this study were: mouse anti-Myc (9E10, Santa Cruz), anti-Flag (M2, Sigma), anti-SmoN (DSHB), anti-En (DSHB), and anti-PI(4)P (Z-P004, Echelon Biosciences); rabbit anti-β-Gal (Cappel), anti-GFP (Clontech), anti-Acetylated tubulin (Sigma), anti-PS1 [30], and rat anti-Ci (2A1, DSHB). Affinity-purified secondary antibodies (Jackson ImmunoResearch) for multiple labeling were used. Fluorescence signals were acquired on an Olympus confocal microscope and images processed with Olympus Fluoview Ver.1.7c. About 15 imaginal discs were screened and three to five disc images were taken for each genotype.

## Supporting Information

S1 Data Numerical data used in preparation of Figs 1C–1F, 7B, 7C, 7E–7G, S3A–S3D, S4E, S4F, S6A and S7A. (XLSX)

S1 Fig Inactivation of Stt4 and Sac1 by RNAi modifies the Hh phenotypes in the wing and larva. (A) A WT adult wing showing interveins 1–5. (B–C) Wings from flies expressing Stt4RNAi (B) or Sac1RNAi (C) by *C765*-Gal4 do not exhibit phenotypes. (D) A wing from flies expressing Smo^PKA12^ by *C765*-Gal4. Arrow indicates a reproducible wing phenotype with partial fusion between Vein 3 and Vein 4, a phenotype indicating the partial loss of Hh signaling activity. (E) A wing from flies coexpressing Smo^PKA12^ with Stt4RNAi by *C765*-Gal4. Arrowheads indicate enhanced fusion between Vein 3 and Vein 4. (F) A wing from flies coexpressing Smo^PKA12^ with Sac1RNAi by *C765*-Gal4. Arrow indicates the weakened fusion between Vein 3 and Vein 4. (G) Cuticle prep with a WT larva shows the well-organized abdominal cuticles. Arrows indicate the cuticle lines. (H) Larva with *prd*-Gal4 driven Sac1 RNAi shows cuticle lines similar to WT. Arrows indicate the cuticle lines. (I) Larva with *prd*-Gal4 driven Hh RNAi shows the loss of cuticles. Arrow indicates where the cuticles are supposed to be located. (J) Larva with *prd*-Gal4 driven Hh RNAi combined with Sac1 RNAi shows partially recovered cuticles. Arrows indicate the cuticle lines rescued by Sac RNAi.
(TIF)

S2 Fig The overexpression of constitutively active forms of Ci or Smo induces the accumulation of PI(4)P in vivo. (A–C) WT stage 11 embryos were stained for En, Smo, PI(4)P, and Ci, respectively. En staining indicates the Hh expression domain. The domain of Smo accumulation correlates the domain of PI(4)P accumulation. Arrows in C indicate PI(4)P accumulation. (D) A wing disc from flies expressing HA-Ci^-3P^, a constitutively active form of Ci, was immunostained for PI(4)P and HA. Arrow indicates the elevated accumulation of PI(4)P in the wing disc. Shown on the right is an enlarged image indicating the accumulation of PI(4)P. (E) A wing disc from flies expressing Myc-Smo^SD123^, a constitutively active form of Smo, was immunostained for PI(4)P and Myc. Arrow indicates the elevated accumulation of PI(4)P in the wing disc.
(TIF)

S3 Fig PI(4)P treatment elevates Hh signaling activity monitored by the *ptc*-luc reporter. (A) Left panel, S2 cells were treated with PI(4)P at the indicated time points and assayed for *ptc*-luc activity. PI(4)P treatment for 6 h induces the peak Hh signaling activity. Right panel, to monitor phospholipids delivery, ELISA assay with the anti-PI(4)P or anti-PI(4,5)P2 antibody was used. *P < 0.01. † no statistical difference detected. (B) S2 cells in 6-well plates were cotransfected with Myc-Smo^WT^ and different amounts of Hh cDNA and assayed for *ptc*-luc activity. Empty UAST vector was used as a transfection control, and carrier 3 was used as a treatment control. PI(4)P consistently increased the low and high Hh signaling activity. (C) In left panel, the efficiency of RNAi knockdown was monitored by Real-Time PCR. * p < 0.05 versus control (GFP dsRNA). In middle and right panels, RNAi efficiency was also confirmed by knocking down the expression of the transfected constructs in S2 cells. (D) The efficiency of GFP RNAi. * p < 0.05 versus control. GFP RNAi efficiency was monitored by either Real-Time PCR (left panel) or western blot with the anti-GFP antibody (right panel). The underlying data of panels A–D can be found in S1 Data.
(TIF)

S4 Fig PH domain fusion at the C-tail or the third intracellular loop compromised Smo activity. (A) Illustrations of SmoPH and SmoL3PH are shown in the left panel. S2 cells were transfected with Myc-SmoPH or Myc-SmoL3PH and immunoprecipitated with the anti-Myc antibody to enrich Smo protein. Purified Smo proteins were incubated with PI(4)P beads to examine the interaction between PI(4)P and Myc-Smo (shown in the right panel). The high mobility shift of SmoL3PH, compared to SmoPH, was due to the additional PH domain inserted. (B–B”) A wing disc from flies expressing Myc-Smo^WT^ was immunostained for Ci and Myc. An enlarged view in B” shows the localization of Myc-Smo. Arrow indicates the elevated Ci staining induced by the expression of Myc-Smo^WT^. (C–C”) A wing disc expressing Myc-SmoPH was immunostained for Ci and Myc. An enlarged view in C” shows the localization of Myc-SmoPH, which had no obvious difference compared to Myc staining in B”. Arrow indicates the less elevated Ci staining compared to B. (D–D”) A wing disc from flies expressing Myc-SmoL3PH was immunostained for Ci and Myc. An enlarged view in D” shows the localization of Myc-Smo. Arrow indicates Smo accumulation in punctate dots, compared to B” and C”. Of note, Myc-SmoL3PH does not induce any Ci elevation in D. (E) S2 cells were transfected with the indicated constructs, treated with either carrier 3 or carrier 3 plus PI(4)P, and assayed for the *ptc*-luc reporter activity. Empty UAST vector served as transfection control. SmoPH and SmoL3PH have little to no *ptc*-luc reporter activity. PI(4)P enhances the activity of Smo^WT^ and Smo^SD123^ but not the activity of Smo^RA4^ and Smno^SD123RA4^. *p < 0.05 versus Smo^WT^ treated with carrier. † *p* < 0.05 versus Smo^WT^ treated with PI(4)P. ** *p* < 0.01 versus Smo^SD123^ treated with carrier. †† *p* < 0.01 versus Smo^SD123^ treated with PI(4)P. *** *p* < 0.01 versus Smo^WT^ treated with carrier. ††† *p* < 0.01 versus Smo^WT^ treated with PI(4)P. **** *p* < 0.01 versus Smo^WT^ treated with carrier. †††† *P* < 0.01 versus Smo^WT^ treated with PI(4)P. (F) FRET efficiency from the indicated WT or mutant Smo with CFP or YFP tagged to their C-terminus. S2 cells were transfected with the indicated constructs, treated with Hh-conditioned medium or control medium, carrier 3 or carrier 3 plus PI(4)P, and assayed for FRET. Smo^RA4^ have significantly reduced FRET value compared to Smo^WT^ (* *p* < 0.01 versus Smo^WT^), and have much less responsiveness to Hh and PI(4)P stimulation († *p* < 0.01 versus Smo^WT^ treated with Hh; †† *p* < 0.01 versus Smo^WT^ treated with PI(4)P). (G) Image enlarged from Fig 3M, with arrow indicating the clone and yellow line indicating the A/P boundary defined by the Ci staining (Blue). The underlying data of panels E–F can be found in S1 Data.
(TIF)

S5 Fig Gprk2 regulates the levels of PI(4)P in wing disc. (A) A wing disc expressing Gprk2RNAi by *ap*-Gal4 was stained for PI(4)P and Ci. GFP indicates RNAi domain of the dorsal compartment. Arrow indicates the decreased accumulation of PI(4)P caused by knockdown of Gprk2. (B) A wing disc expressing RFP-PH^Gprk2^ by *MS1096-*Gal4 was stained for PI(4)P and Ci. Overexpression of the PH domain of Gprk2 increases the level of PI(4)P (arrow). (C) A wing disc expressing RFP-PH^Gprk2^ by *MS1096-*Gal4 was stained for Smo. Arrow indicates the increased accumulation of Smo by the overexpression of the PH domain of Gprk2.
(TIF)

S6 Fig Hh does not regulate the transcription and protein stability of Stt4 and Sac1. (A) The levels of Stt4 and Sac1 mRNA were monitored by Real-Time PCR when S2 cells were treated with 60% Hh-conditioned medium or 60% conditioned medium plus Hh cDNA transfection (to achieve the highest levels of Hh activity). No statistical differences detected. (B–C) Wing discs expressing Flag-Stt4 or GFP-Sac1 by *MS1096*-Gal4 were stained for Flag and GFP. A/P boundary was defined by Ci staining. There were no Flag and GFP staining differences between A and P compartments, indicating Hh does not regulate the stability of the protein. The underlying data of panel A can be found in S1 Data.
(TIF)

S7 Fig Shh activity promotes the production of PI(4)P in NIH3T3 cells. (A) Left panel, NIH3T3 cells were treated with the indicated combinations of Shh, Ptc1 dsRNA, and cyclopamine, a potent Smo antagonist. PI(4)P was detected by ELISA. * *p* < 0.001 versus control (first column); ** no statistical difference detected versus control (first column); † no statistical differences detected versus cyclopamine treatment alone (fourth column). Right panel, Ptc1 RNAi efficiency in NIH3T3 cells was shown by western blot with the anti-Myc-antibody to detect the transfected Myc-Ptc1, and shRNA1 was used in this study. (B) NIH3T3 cells transfected with YFP-Ptc1 or GFP-Ptc2 were immunostained to show the expression of Acetylated (Ac)-tubulin (red; primary cilium), YFP (green; Ptc1), and GFP (green; Ptc2). Images in the inserts are enlarged views with shifted overlays to show the ciliary localization of Ptc1 or Ptc2. About 100 ciliated cells were counted for each set. Quantification of ciliary localization of Ptc1 or Ptc2 is shown in Fig 7G in the main text. The underlying data of panel A can be found in S1 Data.
(TIF)

S1 Methods Additional methods. We provide additional information for the generation of various constructs, transgenic lines, and mutants. We also describe the procedures for GST fusion protein purification and in vitro kinase assay, luciferase assay, and cuticle preparation.
(DOCX)

S1 Table RNAi screen for PITP involved in Smo regulation by PI(4)P. The results and phenotypes from knockdown of the indicated PITP by RNAi using different Gal4 lines are shown in the table. Cell culture assays for Smo stability and phosphorylation are also shown with the primers used for the synthesis of individual dsRNA.
(DOCX)

## Abbreviations

A: anterior
aPKC: atypical PKC
Ci: cubitus interruptus
CK1: casein kinase 1
CK2: casein kinase 2
Cos2-Fu: Costal2-Fused
CRD: cysteine rich domain
C-tail: C-terminal tail
dpp: decapentaplegic
ELISA: enzyme-linked immunosorbent assay
en: engrailed
FRET: fluorescence resonance energy transfer
GPCR: G protein-coupled receptor
Gprk2: G protein-coupled receptor kinase 2
GST: glutathione S-transferase, Hh, Hedgehog
MEF: mouse embryonic fibroblast
MRM: multiple reaction monitor
mSmo: mouse Smo
OSBP: oxysterol binding protein
P: posterior
PA: phosphatidic acid
PH: pleckstrin homology
PI: phosphatidylinositol
PI(4)P: phosphatidylinositol 4-phosphate
PITP: PI(4)P transport protein
PKA: protein kinase A
PS: phosphatidylserine
Ptc: Patched
Ptc-Ihog: Patched-Interference Hh
RND: resistance, nodulation, division
ShhNp: sonic Hh N-terminus
Smo: Smoothened
SSD: sterol-sensing domain
VDRC: Vienna *Drosophila* Resource Center
