# Supplementary figures and images for: PI(4)P Promotes Phosphorylation and Conformational Change of Smoothened through Interaction with Its C-terminal Tail
Source: PLoS Biol. 2016 Feb 10;14(2):e1002375. doi: 10.1371/journal.pbio.1002375 (PMC4749301; doi:10.1371/journal.pbio.1002375)

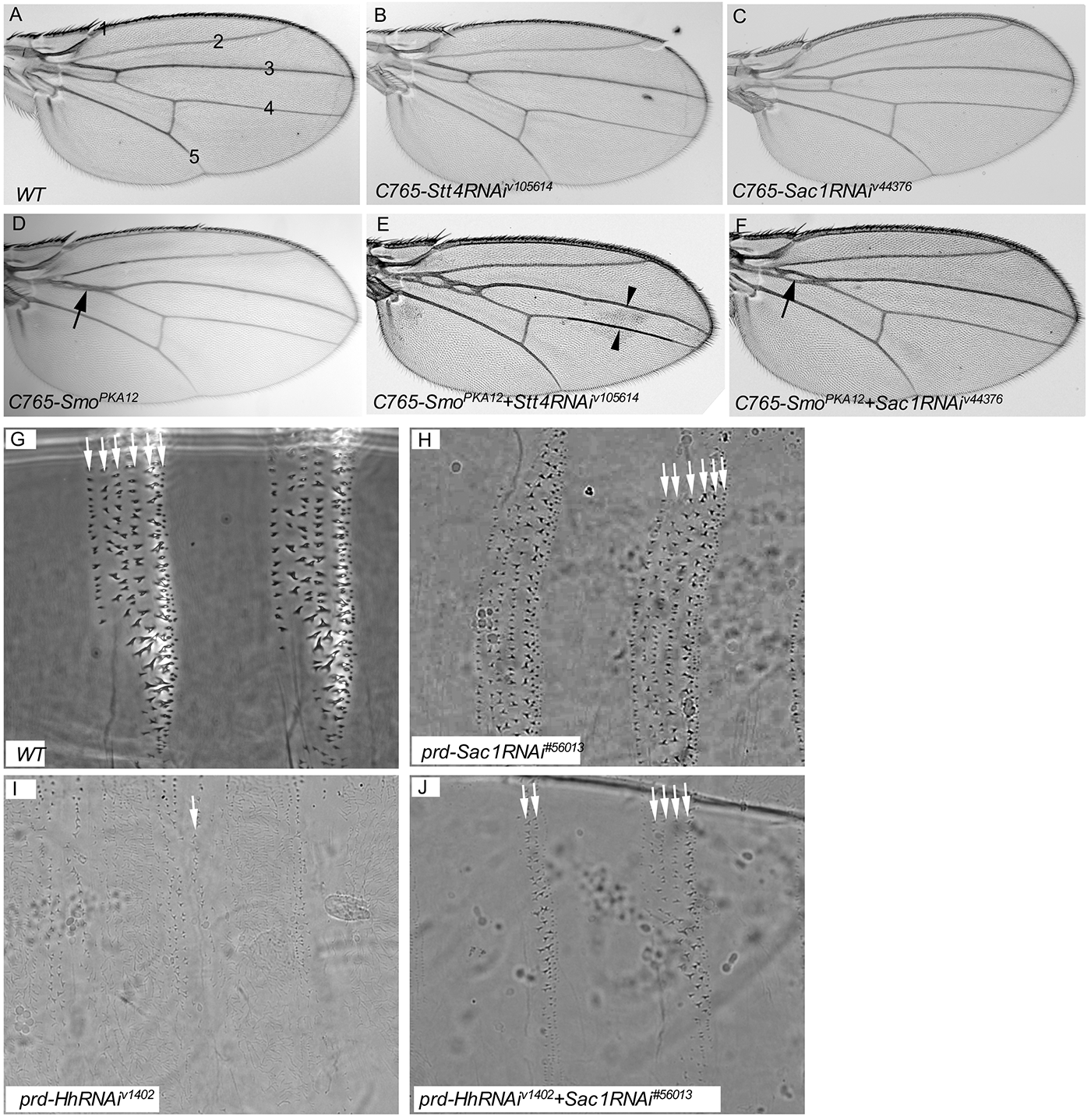

Supplement: S1 Fig — (A) A WT adult wing showing interveins 1–5. (B–C) Wings from flies expressing Stt4RNAi (B) or Sac1RNAi (C) by C765-Gal4 do not exhibit phenotypes. (D) A wing from flies expressing SmoPKA12 by C765-Gal4. Arrow indicates a reproducible wing phenotype with partial fusion between Vein 3 and Vein 4, a phenotype indicating the partial loss of Hh signaling activity. (E) A wing from flies coexpressing SmoPKA12 with Stt4RNAi by C765-Gal4. Arrowheads indicate enhanced fusion between Vein 3 and Vein 4. (F) A wing from flies coexpressing SmoPKA12 with Sac1RNAi by C765-Gal4. Arrow indicates the weakened fusion between Vein 3 and Vein 4. (G) Cuticle prep with a WT larva shows the well-organized abdominal cuticles. Arrows indicate the cuticle lines. (H) Larva with prd-Gal4 driven Sac1 RNAi shows cuticle lines similar to WT. Arrows indicate the cuticle lines. (I) Larva with prd-Gal4 driven Hh RNAi shows the loss of cuticles. Arrow indicates where the cuticles are supposed to be located. (J) Larva with prd-Gal4 driven Hh RNAi combined with Sac1 RNAi shows partially recovered cuticles. Arrows indicate the cuticle lines rescued by Sac RNAi. (TIF) [file pbio.1002375.s002.tif]

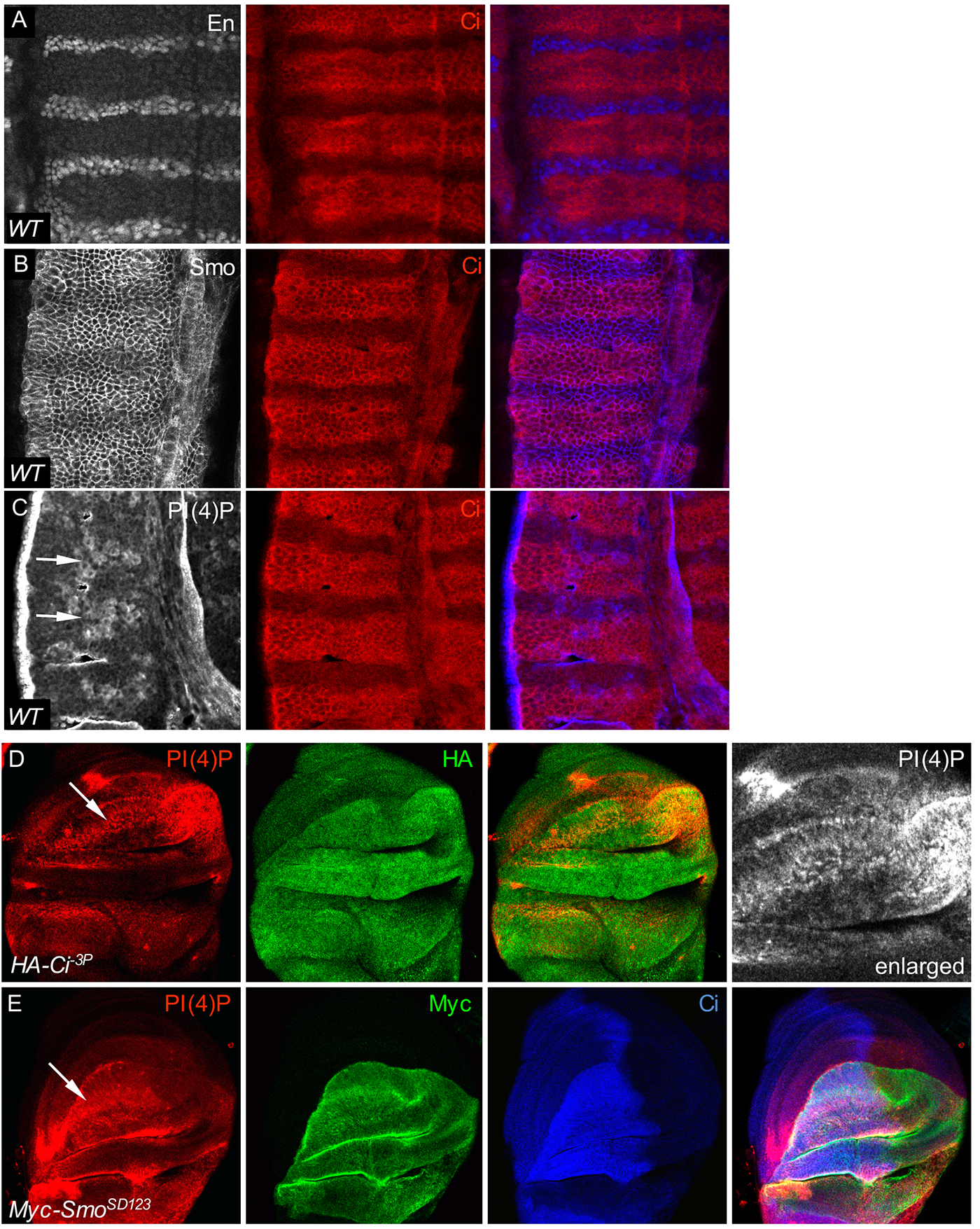

Supplement: S2 Fig — (A–C) WT stage 11 embryos were stained for En, Smo, PI(4)P, and Ci, respectively. En staining indicates the Hh expression domain. The domain of Smo accumulation correlates the domain of PI(4)P accumulation. Arrows in C indicate PI(4)P accumulation. (D) A wing disc from flies expressing HA-Ci-3P, a constitutively active form of Ci, was immunostained for PI(4)P and HA. Arrow indicates the elevated accumulation of PI(4)P in the wing disc. Shown on the right is an enlarged image indicating the accumulation of PI(4)P. (E) A wing disc from flies expressing Myc-SmoSD123, a constitutively active form of Smo, was immunostained for PI(4)P and Myc. Arrow indicates the elevated accumulation of PI(4)P in the wing disc. (TIF) [file pbio.1002375.s003.tif]

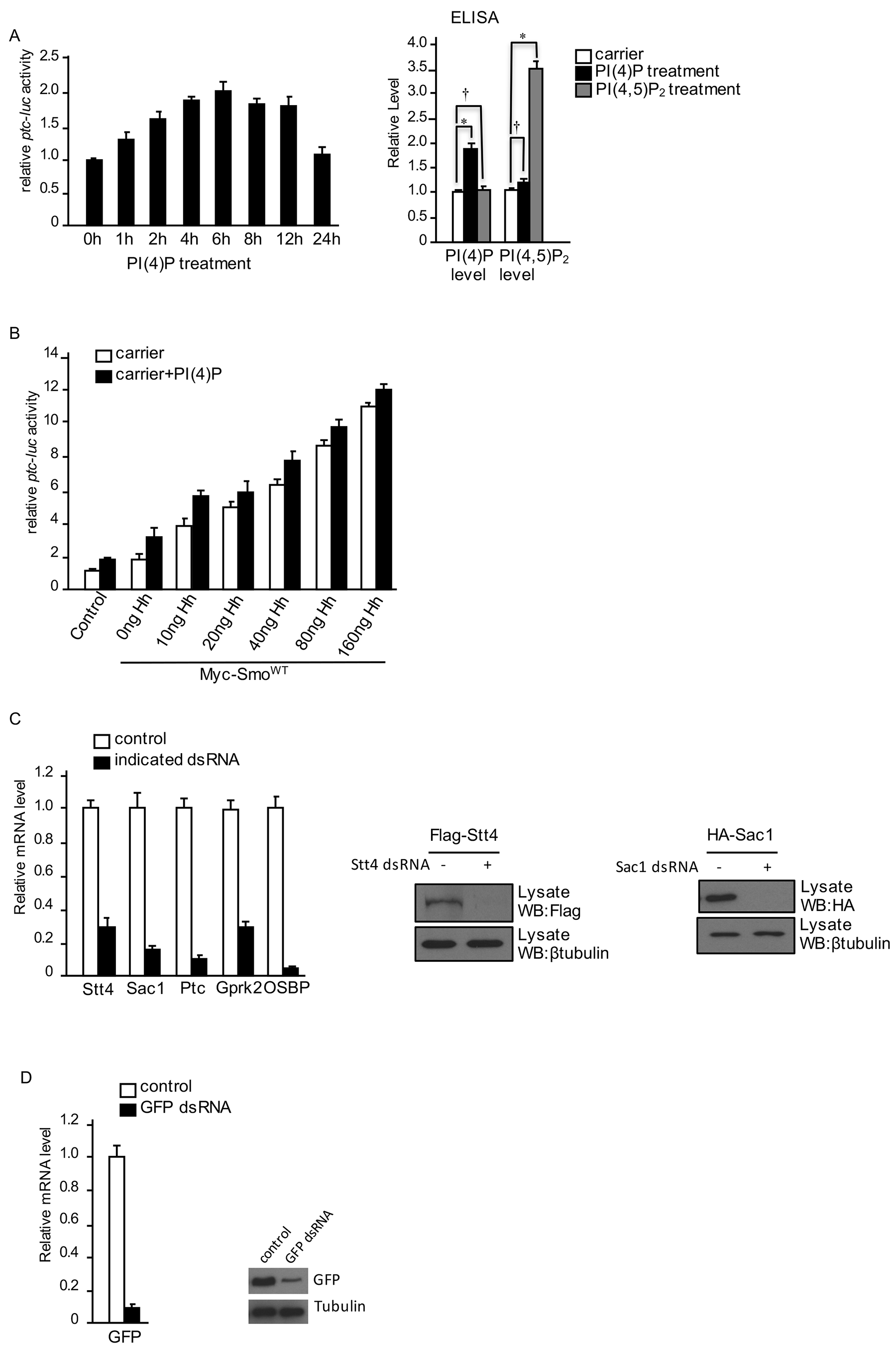

Supplement: S3 Fig — (A) Left panel, S2 cells were treated with PI(4)P at the indicated time points and assayed for ptc-luc activity. PI(4)P treatment for 6 h induces the peak Hh signaling activity. Right panel, to monitor phospholipids delivery, ELISA assay with the anti-PI(4)P or anti-PI(4,5)P2 antibody was used. *P < 0.01. † no statistical difference detected. (B) S2 cells in 6-well plates were cotransfected with Myc-SmoWT and different amounts of Hh cDNA and assayed for ptc-luc activity. Empty UAST vector was used as a transfection control, and carrier 3 was used as a treatment control. PI(4)P consistently increased the low and high Hh signaling activity. (C) In left panel, the efficiency of RNAi knockdown was monitored by Real-Time PCR. * p < 0.05 versus control (GFP dsRNA). In middle and right panels, RNAi efficiency was also confirmed by knocking down the expression of the transfected constructs in S2 cells. (D) The efficiency of GFP RNAi. * p < 0.05 versus control. GFP RNAi efficiency was monitored by either Real-Time PCR (left panel) or western blot with the anti-GFP antibody (right panel). The underlying data of panels A–D can be found in S1 Data. (TIF) [file pbio.1002375.s004.tif]

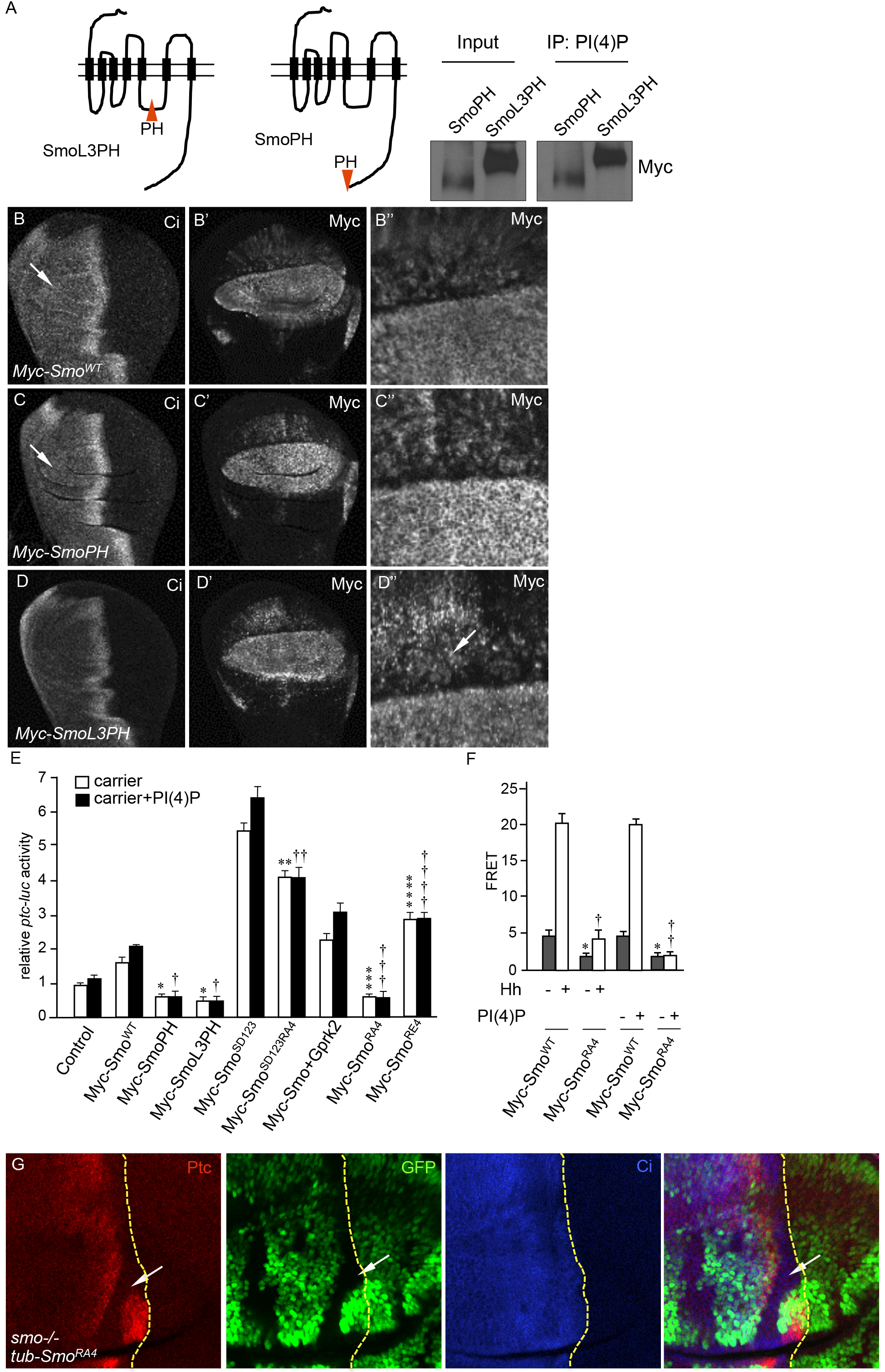

Supplement: S4 Fig — (A) Illustrations of SmoPH and SmoL3PH are shown in the left panel. S2 cells were transfected with Myc-SmoPH or Myc-SmoL3PH and immunoprecipitated with the anti-Myc antibody to enrich Smo protein. Purified Smo proteins were incubated with PI(4)P beads to examine the interaction between PI(4)P and Myc-Smo (shown in the right panel). The high mobility shift of SmoL3PH, compared to SmoPH, was due to the additional PH domain inserted. (B–B”) A wing disc from flies expressing Myc-SmoWT was immunostained for Ci and Myc. An enlarged view in B” shows the localization of Myc-Smo. Arrow indicates the elevated Ci staining induced by the expression of Myc-SmoWT. (C–C”) A wing disc expressing Myc-SmoPH was immunostained for Ci and Myc. An enlarged view in C” shows the localization of Myc-SmoPH, which had no obvious difference compared to Myc staining in B”. Arrow indicates the less elevated Ci staining compared to B. (D–D”) A wing disc from flies expressing Myc-SmoL3PH was immunostained for Ci and Myc. An enlarged view in D” shows the localization of Myc-Smo. Arrow indicates Smo accumulation in punctate dots, compared to B” and C”. Of note, Myc-SmoL3PH does not induce any Ci elevation in D. (E) S2 cells were transfected with the indicated constructs, treated with either carrier 3 or carrier 3 plus PI(4)P, and assayed for the ptc-luc reporter activity. Empty UAST vector served as transfection control. SmoPH and SmoL3PH have little to no ptc-luc reporter activity. PI(4)P enhances the activity of SmoWT and SmoSD123 but not the activity of SmoRA4 and SmnoSD123RA4. *p < 0.05 versus SmoWT treated with carrier. † p < 0.05 versus SmoWT treated with PI(4)P. ** p < 0.01 versus SmoSD123 treated with carrier. †† p < 0.01 versus SmoSD123 treated with PI(4)P. *** p < 0.01 versus SmoWT treated with carrier. ††† p < 0.01 versus SmoWT treated with PI(4)P. **** p < 0.01 versus SmoWT treated with carrier. †††† P < 0.01 versus SmoWT treated with PI(4)P. (F) FRET efficiency from the indicated WT or [file pbio.1002375.s005.tif]

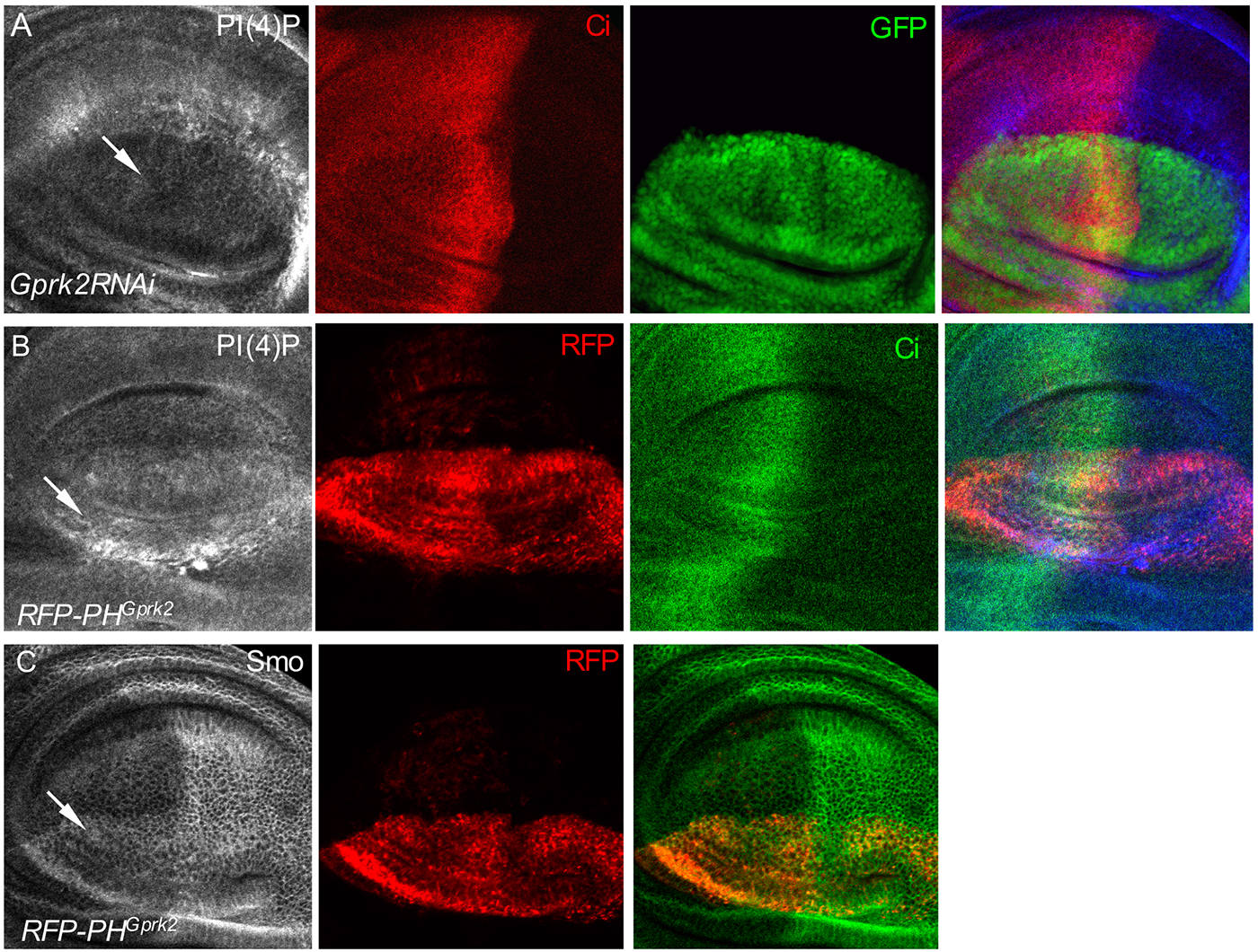

Supplement: S5 Fig — (A) A wing disc expressing Gprk2RNAi by ap-Gal4 was stained for PI(4)P and Ci. GFP indicates RNAi domain of the dorsal compartment. Arrow indicates the decreased accumulation of PI(4)P caused by knockdown of Gprk2. (B) A wing disc expressing RFP-PHGprk2 by MS1096-Gal4 was stained for PI(4)P and Ci. Overexpression of the PH domain of Gprk2 increases the level of PI(4)P (arrow). (C) A wing disc expressing RFP-PHGprk2 by MS1096-Gal4 was stained for Smo. Arrow indicates the increased accumulation of Smo by the overexpression of the PH domain of Gprk2. (TIF) [file pbio.1002375.s006.tif]

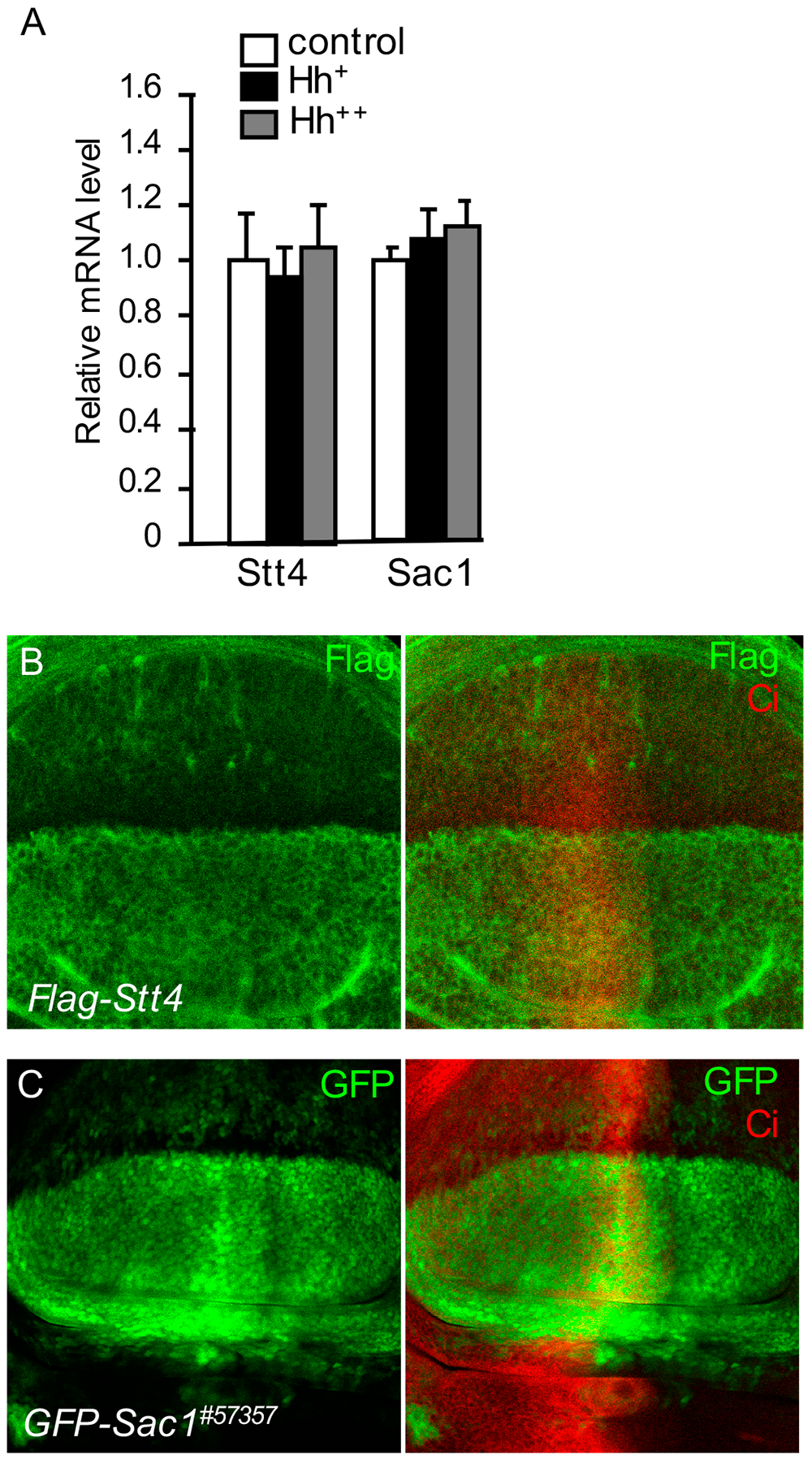

Supplement: S6 Fig — (A) The levels of Stt4 and Sac1 mRNA were monitored by Real-Time PCR when S2 cells were treated with 60% Hh-conditioned medium or 60% conditioned medium plus Hh cDNA transfection (to achieve the highest levels of Hh activity). No statistical differences detected. (B–C) Wing discs expressing Flag-Stt4 or GFP-Sac1 by MS1096-Gal4 were stained for Flag and GFP. A/P boundary was defined by Ci staining. There were no Flag and GFP staining differences between A and P compartments, indicating Hh does not regulate the stability of the protein. The underlying data of panel A can be found in S1 Data. (TIF) [file pbio.1002375.s007.tif]

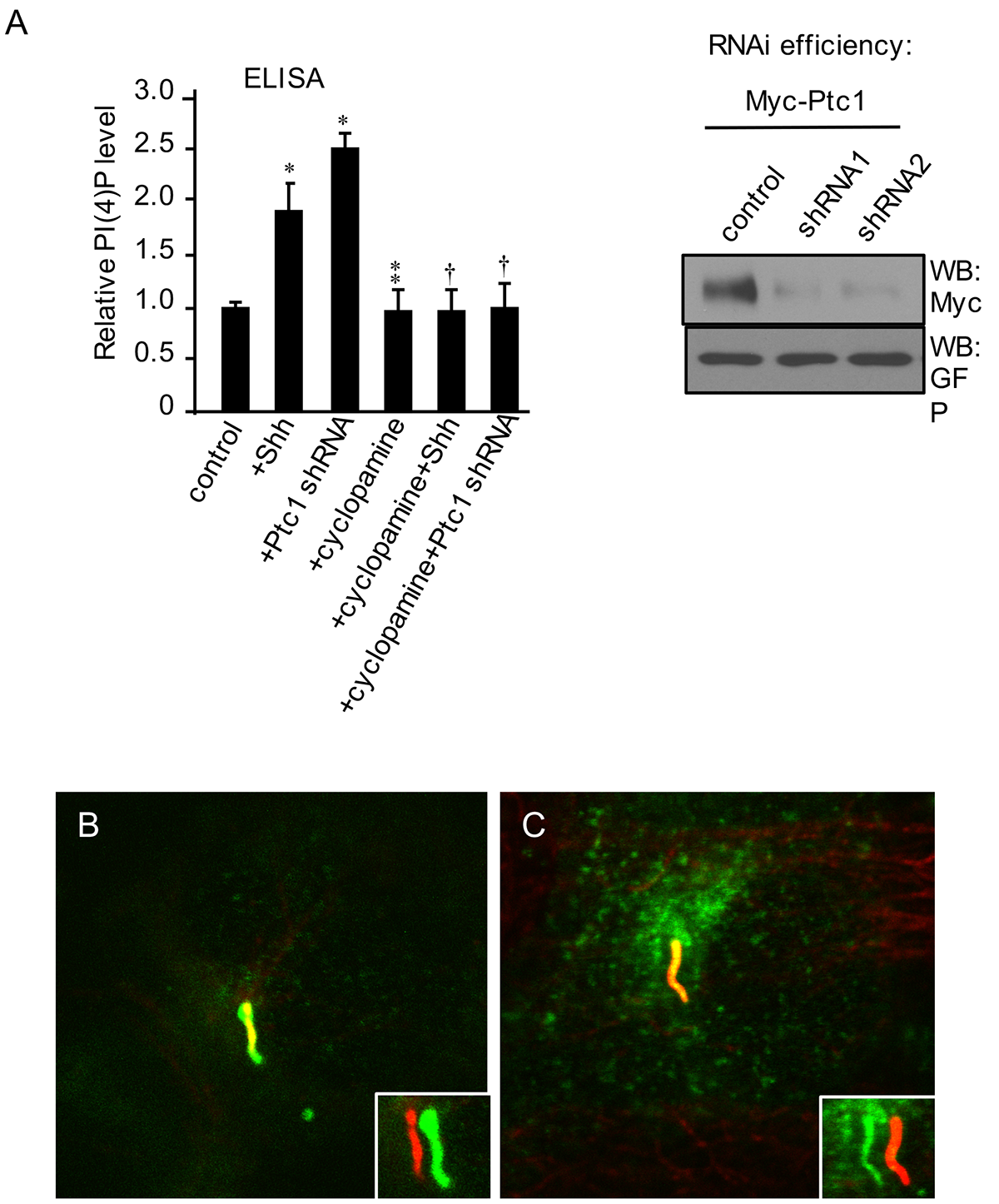

Supplement: S7 Fig — (A) Left panel, NIH3T3 cells were treated with the indicated combinations of Shh, Ptc1 dsRNA, and cyclopamine, a potent Smo antagonist. PI(4)P was detected by ELISA. * p < 0.001 versus control (first column); ** no statistical difference detected versus control (first column); † no statistical differences detected versus cyclopamine treatment alone (fourth column). Right panel, Ptc1 RNAi efficiency in NIH3T3 cells was shown by western blot with the anti-Myc-antibody to detect the transfected Myc-Ptc1, and shRNA1 was used in this study. (B) NIH3T3 cells transfected with YFP-Ptc1 or GFP-Ptc2 were immunostained to show the expression of Acetylated (Ac)-tubulin (red; primary cilium), YFP (green; Ptc1), and GFP (green; Ptc2). Images in the inserts are enlarged views with shifted overlays to show the ciliary localization of Ptc1 or Ptc2. About 100 ciliated cells were counted for each set. Quantification of ciliary localization of Ptc1 or Ptc2 is shown in Fig 7G in the main text. The underlying data of panel A can be found in S1 Data. (TIF) [file pbio.1002375.s008.tif]
