# Supplementary material for: PI(4)P Promotes Phosphorylation and Conformational Change of Smoothened through Interaction with Its C-terminal Tail
Source: PLoS Biol. 2016 Feb 10;14(2):e1002375. doi: 10.1371/journal.pbio.1002375 (PMC4749301; doi:10.1371/journal.pbio.1002375)
Supplement: S1 Methods — We provide additional information for the generation of various constructs, transgenic lines, and mutants. We also describe the procedures for GST fusion protein purification and in vitro kinase assay, luciferase assay, and cuticle preparation. (DOCX) [file pbio.1002375.s009.docx]

**PI(4)P promotes phosphorylation and conformational change of Smoothened through interaction with its C-terminal tail**

**Supplemental Methods**

**GST fusion protein purification and *in vitro* kinase assay**

To examine the effects of PI(4)P on Smo phosphorylation *in vitro*, GST-Smo fusion proteins expressed in bacteria were harvested, washed with PBS, and suspended with lysis buffer at the ratio of 50 µL buffer/1 mL culture. After sonication and centrifugation, glutathione beads were added to the supernatant aliquots (15 µL beads/500 µL lysate), for one hour, followed by three PBS washes at 4°C. The GST fusion protein was then subjected to a kinase assay using commercial PKA and CK1 (New England Biolabs) according to the supplier’s protocols. PtdIns lipids (stock solution 1mM) were added to a final concentration of 10 µM to examine the effect on Smo phosphorylation. Phosphorylation of Smo was detected by western blot with the anti-SmoP antibody [[1](#_ENREF_44)]. For GST-mSmo^608-670^ phosphorylation by Gprk2, Flag-tagged Gprk2 proteins were expressed in S2 cells, immunoprecipitated with the anti-Flag antibody (M2, Sigma), eluted with Flag peptide (Sigma, 1 mg/mL in 50 mM HEPES pH 7.5, 100 mM NaCl, 0.1% NP-40, 5% glycerol), concentrated with centrifugal filter units (Millipore), and incubated with GST-mSmo^608-670^ at 30°C for 1 h in kinase buffer (20mM Tris-HCl at pH 8.0, 2mM EDTA, 10mM MgCl_2_, 1mM DTT, plus 0.1 mM ATP). Phosphorylated GST-mSmo^608-670^ was detected by western blot with the anti-PS1 antibody [[2](#_ENREF_18)].

**Cell culture, immunoprecipitation, western blot, FRET, luciferase reporter assay, and statistical analysis**

For experiments with cultured *Drosophila* S2 cells, transfections were carried out using Effectene transfection reagent (Qiagen). Forty-eight hours post-transfection, cells were treated with lysis buffer followed by centrifugation at 12000 rpm for 10 min. For each sample, 6 × 10^6^ cells were harvested and lysed in 450 µL lysate buffer. 50 µL was saved for direct western blots, with 4 µL for each load. The remaining 400 µL was used for immunoprecipitation assay, generating 30 µL samples. 5 µL immunoprecipitation sample was loaded for each run. For immunoprecipitation, the cell lysate was incubated with the proper primary antibody for 2 hours and added with beads of protein A ultralink resin (Thermo). Protein samples were resolved by SDS-PAGE and transferred onto PVDF membranes (Millipore) for western blot, which was performed using the indicated antibodies and the enhanced chemiluminescence (ECL) protocol. For all the experiments with cultured S2 cells, at least three independent repeats were performed. For all the experiments to quantify PI(4)P, either a stable cell line constitutively expressing *tub*-Ci or S2 cells cotransfection with UAST-Ci construct were used.

To normalize the levels of Smo, 50 μM MG132 and 15 mM NH_4_Cl was used to block Smo degradation, and samples were normalized for loading [[1](#_ENREF_44)]. The use of HhN-conditioned medium has been previously described [[1](#_ENREF_44)]. The method of using MG132 (Calbiochem), a proteasome inhibitor, and NH_4_Cl (Sigma-Aldrich), a lysosome inhibitor, to block Smo degradation has been previously described [[3](#_ENREF_24),[4](#_ENREF_25)].

Treating S2 cell with dsRNA has been described [5,[1](#_ENREF_44)]. dsRNA was synthesized against Stt4 (nucleotides 541-1140), Sac1 (nucleotides 91-690), Ptc (nucleotides 1-781), Gprk2 (nucleotides 1411-2110), Gprk2 3’-UTR (+19 - +533 after stop codon), and OSBP (nucleotides 1412-1976) and RNAi efficiency for each individual RNAi was monitored by quantitative Real-Time PCR (Fig. S3C). GFP dsRNA was synthesized against nucleotides 6-606, and RNAi efficiency was monitored by either RT-PCR or western blot with the anti-GFP antibody to detect the expression levels of cotransfected GFP (Fig. S3C). For Real-Time PCR, total RNA was extracted using Trizol reagent (Invitrogen). cDNA was synthesized using SuperScript III First Strand Synthesis kit (Invitrogen) from 1.0 μg total RNA according to the manufacturer’s instructions. Quantitative Real-Time PCR reactions were carried out using SYBR Green PCR master mix reagents (Thermo) on the ABI StepOnePlus Real-Time PCR System (Applied Biosystems). Thermal cycling was conducted at 95 °C for 30 sec, followed by 40 cycles of amplification at 95 °C for 5 sec, 55 °C for 30 sec and 72 °C for 15 sec. The following primers were used to amplify: Stt4: 5’-TTGACGTCGACTATAATGTG-3’ and 5’-GAGCTCCAGGTTCAGTTTCT-3’; Sac1: 5’-TGAGCAGGATCAGTTCGGTG-3’ and 5’-CCACATGAAGCACTCCCAGC-3’; Ptc: 5’-ATGCTGTGCTTCAATGTGCT-3’ and 5’-CGACGTGGAGAGCATGAACA-3’; Gprk2: 5’-TAATCGAGAAGCAGATACTG-3’ and 5’-CCATGTTGTA AATGTGGAAT-3’; OSBP: 5’-CTGGAGACGAACGAGGAGGA-3’ and 5’-TAGCATCTTGGACCACTTAT-3’; GFP: GFP: 5’-CTGCTGCCCGACAACCACTA-3’ and 5’-CGTCCATGCCGAGAGTGATC-3’; Actin: 5’- GCGTCGGTCAATTCAATCTT -3’ and 5’- AAGCTGCAACCTCTTCGTCA -3’.

FRET analysis has been previously described [[1](#_ENREF_44)]. Briefly, S2 cells transfected with Smo constructs tagged with CFP or YFP at the C-terminus were treated with HhN-conditioned medium or control medium, or with carrier plus PI(4)P or carrier alone. For maximal Hh signal strength, a UAST-Hh construct was also included in the transfection. Cells were then washed with PBS, fixed with 4% formaldehyde for 20 min, and mounted on slides in 80% glycerol. Fluorescence signals were acquired with the 60× objective on an Olympus confocal microscope. CFP was excited at 458 nm wavelength and the emission was collected through a BA 480-495 nm filter. YFP was excited at 514 nm wavelength and the emission was collected through a BA 535-565 nm filter. The CFP signal was obtained once before (BP) and once after (AP) photobleaching YFP using the full power of the 515 nm laser line for 2-3 min at the top half of each cell, leaving the bottom half of the cell as an internal control. The intensity change of CFP was analyzed using the Olympus Fluoview software. The efficiency of FRET was calculated using the formula: FRET% = [(CFP_AP_-CFP_BP_)/CFP_AP_]×100%. Each data set was based on 20 individual cells. In each cell, five regions of interest in the photobleached area were selected for analysis.

To examine Smo-PI(4)P and Ptc-PI(4)P interaction regulated by Hh stimulation, Myc-Smo^WT^ and Myc-Ptc^WT^ were transfected to S2 cells followed by the treatment with Hh-conditioned medium or control medium. Cell extracts were immunoprecipitated with the anti-Myc antibody. The purified Myc-Smo^WT^ and Myc-Ptc^WT^ proteins were eluted by Myc peptide (Sigma, in 500 mM NaCl), and concentrated by the Centrifugal filter units (Millipore). These proteins were then incubated with the PI(4)P beads followed by western blot with the anti-Myc antibody to examine the PI(4)P-bound proteins under different conditions. To examine Smo interaction with PI(4)P packed in liposome, liposomes were prepared by mixing N-biotinyl cap-phosphatidyl­ethanolamine (PE), phosphatidylcholine (PC), phosphatidylinositol (PI), cholesterol, and PI(4)P (all from Avanti Polar Lipids), drying the mixture under nitrogen, and resuspending to a final concentration of 1 mg/mL of total lipid in liposome buffer (50 mM potassium acetate, 1 mM EDTA, 20 mM Hepes-HCl, pH 7.4). Resuspended lipids were sonicated in a bath sonicator until a homogeneous suspension was formed (5 min). Liposomes were incubated for 60 min at 4 °C with purified Myc-tagged proteins, then affinity purified by adding streptavidin-coated agarose resin (Thermo). After washing with liposome buffer, the mixture subjected to western blot to detect PI(4)P bound proteins.

For *ptc-*luc reporter assays, S2 cells were cultured in 6-well plates and transfected with 50 ng *tub*-Ci and 150 ng *ptc-*luc reporter constructs. 48 h post-transfection, cells were lysed for luciferase activity analysis using the Dual-Luciferase Reporter Assay System (Promega). Renilla was used to normalize the luciferase activity. The measurements of Dual-Luciferase were performed using a GLOMAX Multi Detection System (Promega). *Gli*-luc was performed using NIH3T3 cells with the *8*×*GliBS*-luc reporter construct (8×*Gli-mut*-luc construct as a control) and a control pRL-TK. Presented data are representative of three assays, with standard deviation (SD) bars indicated from four replicates. For statistical analysis, Student’s *t* test was used for cell culture studies involving two independent groups.

For PtdIns delivery, PI(4)P (Echelon Biosciences, P4008), PI(4,5) P_2_ (Echelon Biosciences, P4508), PI (Echelon Biosciences, P0008), and PI(3,4,5) P_3_ (Echelon Biosciences, P3908) were delivered intracellularly via Shuttle PIP^TM^ carriers 3 (Echelon Biosciences, P-9C3) or 2 (Echelon Biosciences, P-9C2). PtdIns lipids were pre-mixed with their respective carrier at 1:1 molar ratio at room temperature for 10 min and then added to cells at a final concentration of 10 µM (the same concentration used for S2 and NIH3T3 cells). Cells were then incubated for 2 h before the assays. The delivery of PI(4)P and PI(4,5)P_2_ in S2 cells and the delivery of PI(4)P in NIH3T3 cells were monitored by ELISA assay with the anti-PI(4)P and anti-PI(4,5)P_2_ antibodies.

Antibodies used in this study were: mouse anti-Myc (9E10, Santa Cruz), anti-Flag (M2, Sigma), anti-HA (F7, Santa Cruz), anti-GFP (Millipore); rabbit anti-SmoP [[1](#_ENREF_44)], anti-PS1 [6], and anti-GST (Santa Cruz).

For Ptc RNAi in NIH3T3 cells, the cDNA target sequences of shRNAs for mPtch1 were 5’-GGCGCTAATGTTCTGACCACA-3’ and 5’-TGTTCTGACCACAGAGGCTCT-3’, and were inserted into pLKO.1 (gift from Dr. Tianyan Gao). RNAi efficiency was monitored by western blot with the anti-Myc antibody to detect the expression levels of cotransfected Myc-mPtch1.

***In vitro* kinase assay of Stt4 and phosphatase assay of Sac1**

S2 cells were transfected with Flag-Stt4 and treated with Hh-conditioned medium or control medium. Immunoprecipitates were eluted by Flag peptides (Sigma), and concentrated by Centrifugal filter units (Millipore) followed by *in vitro* kinase assay as described [7]. Briefly, the reactions were performed in 50 μL kinase buffer (25 mM Tris-HCl, pH 7.4, 5 mM MgCl_2_, 1 mM EDTA, 0.1 mM EGTA, 1 mM DTT), containing 1 mg of protein. Samples were mixed with a sonicated solution of 70 μM phosphatidylinositol, and 35 μM phosphatidylserine (Avanti Polar Lipids) and pre-incubated for 20 min at 25 °C. Reaction was started by adding γ-[^32^P] ATP (5 μCi/assay). After 15 min, the reactions were stopped by adding 0.3 mL of methanol, 1 M HCl (1:1, v/v) and extracted with 0.25 mL of chloroform. The organic layer was dried under nitrogen, re-suspended in chloroform, and chromatographed on oxalate-pretreated TLC silica gel plate (Sigma). The reaction products were visualized by autoradiography.

In the phosphatase assay, S2 cells were transfected with HA-Sac1 and treated with Hh-conditioned medium or control medium. Purified HA-Sac1 proteins were subjected for the phosphatase assay with radioactive PI(4)P as substrate, which was made using PI4K2A (SignalChem) to phosphorylate phosphatidylinositol (PI) as previously described [8], with modifications. Reaction for γ-[^32^P] ATP labeled PI(4)P was performed in 25 μL kinase buffer containing 0.5 μg of PI4K2A. Samples were mixed with a sonicated solution of 300 μM phosphatidylinositol and 150 μM phosphatidylserine, and pre-incubated for 20 min at 30 °C. Reaction was started by adding 10 μCi of γ-[^32^P] ATP for 15 min. The reactions were stopped and lipids were extracted as described above. Then the phosphatase assays were performed in 50 μL phosphatase buffer (25 mM Hepes, pH 7.4, 0.1 mM EDTA, 5 mM DTT, 100 μg/mL BSA), containing 1 mg of protein and 100 μM radioactive PI(4)P. After 15 min at 30 °C, reactions were stopped. Lipids were extracted, resuspended after drying under nitrogen, and chromatographed on TLC plate following by autoradiography as described above.

**Cuticle preparation**

Make a 0-6 h embryo collection and incubate for 24-36 h at 25 **°**C, dechorionate the embryos, transfer embryos to 1.5 mL tube and wash with 100% methanol twice. Transfer embryos to slide, remove methonal, place 25-50 µL Hoyer’s Mount Medium and put on cover slip.

**References**

1. Fan J, Liu Y, Jia J. Hh-induced Smoothened conformational switch is mediated by differential phosphorylation at its C-terminal tail in a dose- and position-dependent manner. Dev Biol. 2012;366(2):172-84. Epub 2012/04/28. PubMed PMID: 22537496.
2. Chen Y, Li S, Tong C, Zhao Y, Wang B, Liu Y, et al. G protein-coupled receptor kinase 2 promotes high-level Hedgehog signaling by regulating the active state of Smo through kinase-dependent and kinase-independent mechanisms in Drosophila. Genes Dev. 2010;24(18):2054-67. Epub 2010/09/17. PubMed PMID: 20844016.
3. Li S, Chen Y, Shi Q, Yue T, Wang B, Jiang J. Hedgehog-regulated ubiquitination controls smoothened trafficking and cell surface expression in Drosophila. PLoS Biol. 2012;10(1):e1001239. Epub 2012/01/19. PubMed PMID: 22253574; PubMed Central PMCID: PMC3254653.
4. Xia R, Jia H, Fan J, Liu Y, Jia J. USP8 promotes smoothened signaling by preventing its ubiquitination and changing its subcellular localization. PLoS Biol. 2012;10(1):e1001238. Epub 2012/01/19. PubMed PMID: 22253573; PubMed Central PMCID: PMC3254663.
5. Jia H, Liu Y, Yan W, Jia J. PP4 and PP2A regulate Hedgehog signaling by controlling Smo and Ci phosphorylation. Development. 2009;136(2):307-16. Epub 2008/12/18. PubMed PMID: 19088085; PubMed Central PMCID: PMC2685971.
6. Chen Y, Sasai N, Ma G, Yue T, Jia J, Briscoe J, et al. Sonic Hedgehog dependent phosphorylation by CK1alpha and GRK2 is required for ciliary accumulation and activation of smoothened. PLoS Biol. 2011;9(6):e1001083. Epub 2011/06/23. PubMed PMID: 21695114; PubMed Central PMCID: PMC3114773.
7. Oude Weernink PA, Schulte P, Guo Y, Wetzel J, Amano M, Kaibuchi K, et al. Stimulation of phosphatidylinositol-4-phosphate 5-kinase by Rho-kinase. Journal Of Biological Chemistry. 2000;275(14):10168-74. PubMed PMID: 10744700.
8. Damen JE, Liu L, Rosten P, Humphries RK, Jefferson AB, Majerus PW, et al. The 145-kDa protein induced to associate with Shc by multiple cytokines is an inositol tetraphosphate and phosphatidylinositol 3,4,5-triphosphate 5-phosphatase. Proceedings Of The National Academy Of Sciences Of The United States. 1996;93(4):1689-93. PubMed PMID: 8643691; PubMed Central PMCID: PMCPMC40003.
