# Supplementary material for: PI(4)P Promotes Phosphorylation and Conformational Change of Smoothened through Interaction with Its C-terminal Tail
Source: PLoS Biol. 2016 Feb 10;14(2):e1002375. doi: 10.1371/journal.pbio.1002375 (PMC4749301; doi:10.1371/journal.pbio.1002375)
Supplement: S1 Table — The results and phenotypes from knockdown of the indicated PITP by RNAi using different Gal4 lines are shown in the table. Cell culture assays for Smo stability and phosphorylation are also shown with the primers used for the synthesis of individual dsRNA. (DOCX) [file pbio.1002375.s010.docx]

S1 Table PITPs screened

| CG number  /Gene name | Close mammalian ortholog | Protein function | RNAi lines tested  (v: from VDRC;  #: from BDSC) | Phenotype of adult wing from MS1096-RNAi | Phenotype of wing from C765-SmoPKA12+RNAi | Smo, ptc-lacZ, Ci staining of wing disc from MS1096-RNAi | Oligo for RNAi (none of the RNAi change Smo level and phosphorylation in S2 cells) |
| --- | --- | --- | --- | --- | --- | --- | --- |
| CG5269  /gio(vib) | PITPalpha, PITPbeta | PIP transporter; PI binding | unavailable |  |  |  | **Forward:** GAT CTA GTC TCC GAC GTG GAG AGT GTC CAT AGT TAT ATT CAA GCA TAT CGA CAC TCT GCA CGT CGG;  **Reverse:** CTA GAG CCT CCG ACG TGC AGA GTG TCG ATA TGC TTG AAT ATA ACT ATG GAC ACT CTC CAC GTC GGA |
| CG17818  /rdgBbeta | PITP cytoplasmic 1 | PI transporter | v19089 | No phenotype | No modification | WT staining | **Forward:** GAT CTA GTC CTC ATC GAA GGC TAT GTC GAT AGT TAT ATT CAA GCA TAT GGA CAT AGC GTT CGA TGA GGG  **Reverse:** CTA GAG CCC TCA TCG AAC GCT ATG TCC ATA TGC TTG AAT ATA ACT ATC GAC ATA GCC TTC GAT GAG GAC |
| CG1111  /rdgB(ota1) | PITP cytoplasmic1, PITPNM1, PITPNM2 | PI transporter | #28796 | No phenotype | No modification | WT staining | **Forward:** GAT CTA GTC AGA ATG GTT CTG GGA CAC CAT AGT TAT ATT CAA GCA TAT CGT GTC CCA CAA CCA TTC TGG  **Reverse:** CTA GAG CCA GAA TGG TTG TGG GAC ACG ATA TGC TTG AAT ATA ACT ATG GTG TCC CAG AAC CAT TCT GAC |
|  |  |  | v6226 | No phenotype  (shrunk wing when with ap-Gal4) | Stronger | WT staining (Also WT staining with ap-Gal4) |  |
| CG9528  /retm | Yeast Sec14 | PI transporter | v44686 | No phenotype (shrunk wing when with ap-Gal4) | No modification | WT staining (Also WT staining with ap-Gal4) |  |
|  |  |  | v44687 | No phenotype | No modification | WT staining |  |
| CG7207  /cert | FAPP1/2 | Ceramide transfer protein | v27914 | No phenotype | Stronger | WT staining |  |
|  |  |  | v103563 | No phenotype | Stronger | WT staining |  |
| CG6708  /OSBP | OSBP | Oxysterol binding protein | v108252 | No phenotype | Stronger | WT staining | **Forward:** GAT CTA GTC GTA GCA GGT TCG CAT GCT GTT AGT TAT ATT CAA GCA TAA GAG CAT GCG TAC CTG CTA CGG CT  **Reverse:** CTA GAG CCG TAG CAG GTA CGC ATG CTC TTA TGC TTG AAT ATA ACT AAC AGC ATG CGA ACC TGC TAC GAC TA |
| CG3002  /GGA | GGA1 | PI(4)P adaptor | v3269 | No phenotype | No modification | WT staining |  |
|  |  |  | v3270 | No phenotype | Much stronger | WT staining |  |
| CG9113  /AP-1gamma | AP-1, Gamma adaptin | PI(4)P adaptor | v3275 | Sick wing | Much stronger | Smo Ci ptc-lacZ changes due to morphology changes |  |
| CG8532  /Lqf |  | Clathrin coats complex | #27522, |  |  | WT staining (UAS-Lqf #25104 also tested, WT staining) |  |
| CG42250  /LqfR | EpsinR-clathrin interactor 1 | Clathrin coats complex | #28987 | No phenotype | No modification | WT staining |  |
|  |  |  | v33799 | No phenotype | No modification | WT staining |  |
|  |  |  | v103719 | Narrow smaller wing | No modification | WT staining |  |
| CG3860 |  | Oxysterol binding protein | v107872 | Wing shrunk in proximal region | Sick wing | WT staining |  |
| CG42668 |  | Oxysterol binding protein |  | No phenotype | No modification | WT staining |  |
| CG1513 | FAPP1/2 | Oxysterol binding protein | v39148 | No phenotype | Stronger | WT staining |  |
|  |  |  | v48600 | No phenotype | No modification | WT staining |  |
| CG42248 | FAPP1/2 |  | v46883 | No phenotype | Stronger | WT staining |  |
|  |  |  | v105096 | No phenotype | Stronger | WT staining |  |
|  |  |  | v110590 | Sick wing (male)  Wing proximal phenotype (female) | Stronger | Smo Ci ptc-lacZ changes due to morphology changes |  |
| CG5077 |  | Oxysterol binding protein |  |  |  |  |  |

Note: Some of the RNAi lines tested with MS1096-Dicer that often produced wing phenotypes however did not cause any changes in Smo, Ci and ptc-lacZ staining.
